# Supplementary material for: Damage-programmable design of metamaterials achieving crack-resisting mechanisms seen in nature
Source: Nat Commun. 2024 Aug 27;15:7373. doi: 10.1038/s41467-024-51757-0 (PMC11349770; doi:10.1038/s41467-024-51757-0)
Supplement: Supplementary file 1 — Supplementary Information [file 41467_2024_51757_MOESM1_ESM.pdf]

Supplementary Information for

Damage-programmable design of metamaterials achieving crack-resisting mechanisms seen in nature

Zhenyang Gao[1,2], Xiaolin Zhang[1,2], Yi Wu[1,2,3,4*], Minh-Son Pham[5], Yang Lu[6], Cunjuan Xia[1,2,3,4], Haowei Wang[1,2,3], Hongze Wang[1,2,3,4,7*]

[1]State Key Labortory of Metal Matrix Composites, Shanghai Jiao Tong University, Shanghai, 200240, China
[2]School of Materials Science and Engineering, Shanghai Jiao Tong University, Shanghai, 200240, China
[3]Institute of Alumics Materials, Shanghai Jiao Tong University (Anhui), Huaibei, 235000, China
[4]Anhui Province Industrial Generic Technology Research Center for Alumics Materials, Huaibei Normal University, Huaibei, Anhui 235000, China
[5]Department of Materials, Imperial College London, London, SW7 2AZ, UK
[6]Department of Mechanical Engineering, University of Hong Kong, Hongkong, 999077, China
[7]Shanghai Key Laboratory of Material Laser Processing and Modification, Shanghai, 200240, China

[*]Corresponding authors, email: eagle51@sjtu.edu.cn, hz.wang@sjtu.edu.cn

This PDF file includes:

Supplementary Note 1 to 11

Supplementary Fig. 1 to 31

Supplementary Table 1 to 5

Other supporting materials for this manuscript include the following:

Supplementary Movie 1 to 2

Supplementary Note 1. Data-driven damage design of DP metamaterial

To address the enduring challenge of lacking an effective systematic design strategy for controlling and resisting fractures in metamaterials, a data-driven DP metamaterial design strategy (Supplementary Fig. 1) is introduced to: (1) spatially engineer the crack path, and (2) construct toughening units that translate crack-resisting mechanisms from nature to mechanical metamaterials. Based on the fracture property distribution across the design space by the crack path engineering and toughening unit designs, a machine-learning-assisted (ML-assisted) DP cell design engine is developed to engineer the fracture of each cell for the metamaterials (Supplementary Fig. 2). Specifically, the design of DP cells is proposed according to the given base cell topology, cell size, and face-fiber angles (Supplementary Fig. 2a). The BCC is selected as the topology of the base cell for demonstration purposes. We determine the distance between the face-fibers as the maximum distance that could form effective deflections of the fracture (0.3 of the cell sizes). The size of the unit cells is preserved as 3 mm throughout this study for comparison purposes, where the radius of the structs is designed as 200 µm according to the fabrication limit. The DP surfaces are constructed with programmed face-fiber angles and combined with the base cells to form the DP cells. Depending on the fracture initiation surface (FIS), the surfaces of each DP cell are decomposed to two primary guiding surfaces (PGSs) and four minor guiding surfaces (MGSs) with the face-fiber angle $\{\theta_{g1}, \theta_{g2}, \theta_{g3}, \theta_{g4}, \theta_{g5}, \theta_{g6}\}$. To model the fracture properties of the DP cells, the fracture angle θ_f, fracture energy G_f, and fracture strength σ_f are calculated:

$$\theta_f = (\theta_L + \theta_R)/2 \tag{S1}$$

$$G_f = \int_0^{\delta_f} F \, d\delta_f \tag{S2}$$

$$\sigma_f = F_f / A \tag{S3}$$

where F is the load applied on the edge of the DP cell during the fracture process, θ_L and θ_R are the fracture angles of the left and right PGSs, respectively, δ_f is the fracture displacement, F_f is the ultimate load of a DP cell, and A represents the area of the fracture load. To validate the proposed data-driven damage-programming metamaterial method in a clear and concise manner, and to focus on the fundamental science of fracture mechanics, this work concentrates on the fracture properties derived from the most commonly studied mode I opening fracture configuration. It should also be noted that the proposed method is adaptable to arbitrary fracture modes by incorporating additional training data as shown in (Supplementary Fig. 22). This flexibility stems from the fact that the output layer structure of the fully connected neural networks (FCNNs) concerning fracture properties is independent of any specific load direction. This independence allows the FCNNs to be successfully trained to represent different and complex modes of fractures with appropriately organized training data. Therefore, validating the effectiveness of the proposed design framework not only supports the currently studied load scenarios but also other complex fracture conditions encountered in practical applications.

A fast prediction tool is required to develop the DP cells with programmed fracture behaviors based on their highly abstracted design parameters to access a large number of possibilities of face-fiber angles' combinations, which is difficult to be accomplished by conventional experimental modeling or finite element analysis (FEA) approaches. The maturing ML technique can interpret the underlying patterns of data without conducting time-consuming numerical simulations[1], and is widely applied to support intelligent designs in different types of industries[2, 3, 4, 5, 6]. Hence, TensorFlow-based[7] FCNNs were

developed (Supplementary Fig. 2b) to establish the underlying relationships between fracture properties and face-fiber angles using a training dataset comprising 900 different DP cells generated by a training sample generation algorithm, where the training input consists of various compositions of face-fiber angles, and the training output includes numerically derived fracture angle (θ_{f0}-θ_{fN}), fracture energy (G_{f0}-G_{fN}), and fracture strength (σ_{f0}-σ_{fN}) according to the simulated stress-strain curves and equations (S1-S3) (also refer to Supplementary Fig. 23 for the training sample generation algorithm and Section 4.3 in "Methods" for FEA). The distribution of the training data composition was designed to incorporate the influence of different face-fiber angles, with PGS angles generated from -90° to 90° at intervals of 30° and MGS angles randomly scattered for each pair of PGS angles, along with 500 additional compositions of purely randomly scattered face-fiber angles to supplement the pre-generated PGS angles. The optimized hyperparameters of the FCNNs for θ_f, G_f, and σ_f predictions are summarized in Supplementary Table 5, obtained through evaluation of various combinations of learning rate, number of neurons per layer, and training epochs, with average test data losses from five training processes and detailed comparisons provided in Supplementary Fig. 24 to Supplementary Fig. 26. The rectified linear activation unit (ReLU) is selected with modifications as the activation functions due to its advantages of handling the vanishing gradient issues[8], where the Leaky ReLU activation function is applied for the training option of the fracture angles due to the potential negative values (see Supplementary Fig. 27 for comparison of different activation functions), where the mean squared error loss function showed a substantial decrease. The detailed accuracy validations for the FCNNs are provided in Supplementary Fig. 4. The prediction errors of the model are recorded using

test dataset comprising 10% randomly selected training data. As the number of training DP cells increased, the prediction errors experienced significant decay and converged to 8.8° for θ_f, 0.34 mJ for G_f, and 0.25 MPa for σ_f. The accuracy of the trained model is further validated by experimental fracture results from 10 randomly generated DP cells, fabricated according to the test configuration shown in Supplementary Fig. 6d. The results indicate that the experimentally measured values fall within the prediction ranges of the model, confirming the reliability of the FCNNs for fabricated DP components.

To design the DP cells with desired functional fracture behaviors, we developed a ML-assisted DP cell deep search algorithm[9] that provides the composition of face-fiber angles $\{\theta_{g1}, \theta_{g2}, \theta_{g3}, \theta_{g4}, \theta_{g5}, \theta_{g6}\}$ that optimizes the user-specified functional fracture properties:

$$\text{To Find:} \{\theta_{g1}, \theta_{g2}, \theta_{g3}, \theta_{g4}, \theta_{g5}, \theta_{g6}\}_i$$

$$\text{Maximize: } f(\sigma_{f,i}, G_{f,i}),$$

$$\text{s.t.: } \theta_{f,i} = \theta_{design}, \; i \in [1, N] \tag{S4}$$

where $\{\theta_{g1}, \theta_{g2}, \theta_{g3}, \theta_{g4}, \theta_{g5}, \theta_{g6}\}_i$, $\sigma_{f,i}$, $\theta_{f,i}$, and $G_{f,i}$ is the composition of face-fiber angles, ML-predicted fracture strength, ML-predicted fracture angle, and ML-predicted fracture energy for the ith sample, respectively, N is the total amount of generated $\{\theta_{g1}, \theta_{g2}, \theta_{g3}, \theta_{g4}, \theta_{g5}, \theta_{g6}\}$, θ_{design} is the desired fracture angle from design, $f(\sigma_{f,i}, G_{f,i})$ is the user-defined combination function of fracture properties to be optimized. In each literation, the algorithm generates n DP cells that possess face-fiber angles confined by the face-fiber ranges $\{R_1, R_2, R_3, R_4, R_5, R_6\}$ provided in the previous iteration, and select the DP cell with the desired best outcome of the ML-predicted fracture properties for user-

defined functional purposes. The selected $\{\theta_{g1}, \theta_{g2}, \theta_{g3}, \theta_{g4}, \theta_{g5}, \theta_{g6}\}$ is extracted to update the middle values to redefine $\{R_1, R_2, R_3, R_4, R_5, R_6\}$ with a refinement coefficient β. Once the ranges reach the design resolution of the face-fiber angles, the optimization process stops, and the optimal DP cell is generated with customized functional fracture properties.

The computational cost of the data-driven damage-programming metamaterial design method encompasses training data gathering, ML fracture predictions, and ML-assisted DP cell generation. The computational time is recorded using a laptop equipped with a 2nd Gen Intel® Core™ i5-12500H processor at 2.50 GHz and 16 GB of random access memory. The average duration for simulating a single training data point is 19 minutes, resulting in a total of 210 hours for the data gathering process required for model training. Note that the gathering of training data is a one-time expense that construct the fracture data to train the model, and does not recur during subsequent uses of the method. The computational costs of ML fracture predictions were recorded as total computational time required for the trained FCNNs to compute θ_f, G_f, and σ_f across a range of DP cell numbers, up to 10^5. Compared to the conventional finite element analysis (FEA) approaches, which require an average computational time for a single DP cell of 19 minutes, the trained FCNNs can predict the fracture properties of a DP cell in 34 µs on average (Supplementary Fig. 5a). These results underline the capability of FCNNs to deliver rapid predictions essential for DP cell generation algorithm and for managing microscopic fracture behaviors. The DP cell generation algorithm uses the FCNNs as fast prediction tool for vast datasets, with a time complexity of $O(n\log_{1/\beta}\pi/\theta_{min})$, where θ_{min} is the resolution of the face-fiber angles, and n is the number of DP cells generated in each search

cycle. In this work, DP cell generation parameters were set at θ_{min} = 2°, β = 0.1, and n = 1000 to balance the computational time and design resolution, where Supplementary Fig. 5b indicates the ML-assisted DP cell generation algorithm using these parameters can produce DP metamaterials comprising 10^5 cells within 12 minutes. This capability supports the development of DP metamaterials with enhanced fracture toughening mechanisms[10] or efficient dissipation of fracture energy[11, 12].

Supplementary Note 2. The crack path engineering algorithm

We developed a crack path control algorithm with two steps to spatially control the crack propagation (Supplementary Fig. 28): (1) function mapping of design spaces; (2) generation of fracture properties of DP cells in each region. The spaces of the unit cells satisfying equation (S5) are selected as the guiding regions, where it is composed of unit cells in the design space with overlaps with the crack geometry.

$$S_g = \{S_{cell} | \exists\, p \in f_{\text{fracture}}, S_{cell} \in S, \text{s.t.}\, p \in S_{cell,i}\} \qquad \text{(S5)}$$

where S_g represents the space of guiding regions, S_{cell} stands for the space of a unit cell within the total design space S, and p represents the points on the pre-engineered fracture geometry f_{fracture}. The correction region S_c, adjacent to S_g, is defined with sizes determined by the crack slope variation, ensuring that fracture geometries with higher variations in crack path are assigned larger correction zones to enhance fracture guiding reliability and reduce failure.

$$L_c(p_{eg}) = \gamma \nabla^2 f_{\text{fracture}}(p_{eg}) \qquad \text{(S6)}$$

where γ is the correction coefficient that determines the magnitude of the correction, p_{eg} is an edge point on the fracture geometry that requires a potential neighboring correction zone, and $L_c(p_{eg})$ is the size of the correction zone for p_{eg}. Once S_g and S_c are defined, the unassigned regions are chosen as the background regions S_b. The fracture angle of cells in S_g is designed to match the crack geometry for effective guiding, while maximizing $G_{f,g}/\sigma_{f,g}$ *with* $\sigma_{f,g} < \sigma_{f,c}$ to confine fracture within the desired geometry exhibiting the earliest fracture and largest deformation. Additionally, the fracture angle of cells in S_c is designed to guide the crack towards the nearest uncracked cell in S_g, maintaining $\sigma_{f,c}$ below $\sigma_{f,b}$ to prevent unintended crack propagation into S_b. Conversely, cells within S_b

are unrestricted in fracture angle and engineered to withstand the highest fracture stress σ_{fmax}, thus impeding fracture entry into S_b.

The crack control requirements for the fracture behaviors of DP cells include: guiding cells positioned within the predetermined crack path, correction cells that realign misoriented crack tips, and background cells engineered as obstacles. The DP cells enabling guiding, correction, and background crack path engineering features are generated according to equations (S7-S13) to fill S_g, S_c, and S_b, respectively.

$$\theta_{g,3D,x}(x,y) = \arctan(df_{3D}(x,y)/dx) \tag{S7}$$

$$\theta_{g,3D,y}(x,y) = \arctan(df_{3D}(x,y)/dy) \tag{S8}$$

$$\theta_{c,3D,x}(x,y) = \arctan(d_z/d_x) \tag{S9}$$

$$\theta_{c,3D,y}(x,y) = \arctan(d_z/d_y) \tag{S10}$$

$$\text{maximize } G_{f,g}/\sigma_{f,g} \text{ s.t.} \sigma_{f,g} < \sigma_{f,c} \tag{S11}$$

$$\text{maximize } \sigma_{f,c} \text{ s.t.} \sigma_{f,c} < \sigma_{f,b,} \theta_f = \theta_c \tag{S12}$$

$$\sigma_{f,b} = \sigma_{fmax} \tag{S13}$$

where

$\theta_{g,3D,x}(x,y)$ and $\theta_{g,3D,y}(x,y)$ are the projections of the fracture angles for guiding cells in 3D design space on *yz* plane and *xz* plane, respectively;

$\theta_{c,3D,x}(x,y)$ and $\theta_{c,3D,x}(x,y)$ are the projections of the fracture angles for correction cells in 3D design space on yz plane and xz plane, respectively;

d_x, d_y, and d_z are the x, y, and z differences between the interested correction cell and its closest unbroken guiding cell, respectively;

$G_{f,g}$ is the fracture energy of the guiding cell;

σ_{fmax} is the maximum fracture energy that could be achieved by the data-driven damage-programmable cells, $\sigma_{f,g}$, $\sigma_{f,c}$, and $\sigma_{f,b}$ are the fracture strength of the guiding, correction, and background cells, respectively.

Supplementary Note 3. Toughening unit designs

Materials in nature typically exhibit three types of crack-resisting mechanisms: crack tip interaction, crack shielding, and reinforcement bridging. Crack tip interactions often involve crack bowing units, which are achieved using small second phase particles with ultrahigh fracture strength compared to the matrix, and crack deflection units, which employ large phases with high fracture strength and deflective properties to induce out-of-plane fracture deflection. Crack shielding is facilitated by specific material phases that can expand the crack opening perpendicular to the crack propagation and blunt the crack tip. Reinforcement bridging is characterized by fiber-like bridges with ultrahigh fracture strength that effectively resist fracture propagation. Inspired by the features of natural toughening units, Type-0 (T0) to Type-3 (T3) DP cells have been developed as building blocks to construct toughening units for crack-resisting mechanisms (refer to Supplementary Fig. 3 and Supplementary Table 2).

The CB phases are designed to against the crack with a forced crack by-pass or cut-through with T1 cells, which are programmed with the maximized fracture strength σ_f without fracture guiding features. T2 is the DP cells that has the largest σ_f that could maintain the maximum fracture angles $\theta_{f\,max}$ that are designed at the edges of the CD phases to ensure effective out-of-plane deflections, while the T1 cells are aligned at the middle of the CD phase to avoid being cut-through. Each shielding unit is engineered with either the positive shielding (PS, $\theta_f = \theta_s$) cells or negative shielding (NS, $\theta_f = -\theta_s$) cells with variety of guiding angle θ_s, so that the crack is guided to opposite directions by adjacent shielding layers to blunt the crack tip. The PS and NS cells are designed with Type-3 (T3) DP cells with a range of engineerable fracture angles and strengths.

Compliance layers filled with base cells engineered between the adjacent pair of shielding layers to provide a buffer region for the complete flip of the fracture angles. The reinforcement bridges are construct with multiple T1 cells with different bridge lengths to resist the crack by propagating through the bridges.

Supplementary Note 4. Designs of crack geometries

The two-dimensional and three-dimensional fracture surfaces are defined in equation (S14-S15):

$$f_{2D}(x) = (H/3)\sin[(2\pi/L)x] + H/2 \quad \text{(S14)}$$

$$f_{3D}(x,y) = -(S/4)[\sin((2\pi/S)x) + \cos((2\pi/S)y)] \quad \text{(S15)}$$

where $f_{2D}(x,y)$ and $f_{3D}(x,y)$ are the functions describing the 2D and 3D crack geometries, L and H is the length and height of the 2D design space, S is the size of the 3D design space. These trigonometric functions were selected to test the mechanical metamaterials due to their large variations of fracture angles ranging from 0-90°, and were validated in Fig. 2 and Supplementary Fig. 7.

Supplementary Note 5. Detailed design information of compact tension specimen

The compact tension configuration is selected to evaluate the fracture properties of the proposed DP metamaterials throughout this study with a test region of 10 × 3 × 11 cells in xyz directions. The specimen with crack tip interactions is designed with CD phases (d_{CD} = 1 cell) to deflect the crack to the trapping regions filled with randomly implanted CB phases (ρ_{CB} = 50%). The crack shielding specimen is composed of shield units with moderately reducing g_{fs} from the crack tip to the sides. Depending on the energy increment required for the variation of the crack orientation and the g_{fs} of the shield units, the encountered fracture could be shielded by different levels of the shield regions, while it should be noted that the proposed method is not limited to the design of the shield regions in this work. The specimen with compound reinforcement bridging is designed with reinforcement bridges that have increasing S_B with maximal corresponding d_B that could trigger multiple reinforcement bridging events (Supplementary Fig. 15 and Supplementary Note 4) to improve the fracture energies with the minimized weight addition.

Supplementary Note 6. Selection of design parameters for different crack-resisting mechanisms.

In this work, we design the specimens with crack tip interactions, crack shielding, and reinforcement bridging based on the study of their fracture-resisting design units. The specimens with crack tip interactions are filled with the crack deflection (CD) phase with d_{CD}= 1 cell, and CB phase with ρ_{CB} = 0.5, where these parameters provide the highest density-normalized fracture energies based on the experimental results in Fig. 3. For the crack shielding specimen, we design the shielding regions with different levels of g_{fs} according to the discussion in Supplementary Note 1. For the reinforcement bridging specimen, it is desired to form maximized number of reinforcement bridging events throughout the test region with minimum addition of reinforcement bridges. Therefore, a gradual increment of the size of reinforcement bridges with maximum bridge distance that could trigger those events is designed according to the results in Supplementary Table 3.

Supplementary Note 7. Theoretical derivations of the fracture energy barriers for crack shielding

The shielding fracture energy is one of the controllable fracture behaviors of the shield units to engineer the shield regions of the crack shielding damage-programmable metamaterials. Based on our studies of the shield units, it is observed that the fracture energies vary with different shielding angles (Fig. 3, Supplementary Fig. 15, Supplementary Fig. 10, and Supplementary Fig. 12). Therefore, different energy increments are required to propagate through a layer of shield units with distinguish shielding angles. To quantitively model such property, we define the shielding fracture energy as the average fracture energy required for a crack tip to propagate through a layer of uniform shield units:

$$g_{fs}(\theta_s) = G_{f,S}(\theta_s)/L \tag{S16}$$

where g_{fs} is the shielding energy, θ_s represents the shielding angle, $G_{f,S}$ is the total fracture energy of a specimen with the shield units designed with uniform θ_s, L is the total number of layers damaged by the crack propagation. With this definition, we define the shield zones of an encountered layer of the crack shielding specimens with different levels of g_{fs} for a given crack tip by:

$$S_{shield\ region}(g_{fs}) = \{S_{shiled\ units}(\theta_s) | g_{fs}(\theta_s) \leq g_{fs}\} \tag{S17}$$

where $S_{shield\ region}(g_{fs})$ is the group of the shield units within the shield region with pre-defined shielding energy g_{fs}, $S_{cell}(\theta_s)$ represents the shield units with the shielding angle θ_s. For the crack shielding specimen evaluated in this study, the shield zones are defined as shown in Supplementary Fig. 29.

To study the shield units with different θ_s, we simulate the stress field and the fracture energy of the shielded crack tip with θ_s = 30º - 70º during the crack propagation through the FEA approach (Fig. 3e, Supplementary Fig. 21, and Supplementary Fig. 19c). In addition to the contents in main text, we observe two θ_s-related phenomena: (1) higher shielding energy $\Delta_S G_{f0}$ is required for the crack propagates through the shielding layers with larger θ_s, which is due to a increased fracture energy of T3 cells with larger fracture angles and a reduced stress intensity at the crack tip; (2) the shielded deflection event is more likely to happen for the shielding layers with θ_s closer to 45º, which is caused by their smaller angle compliances (Supplementary Note 6). Therefore, we model the fracture energy of the specimens with uniform shield units based on a shielding energy term $\Delta_S G_{f0}$ and a shielded deflection term $\Delta_{SD} G_{f0}$:

$$G_{f,S} = G_{f,M} + \alpha_S(\Delta_S G_{f0} + P_{SD}\Delta_{SD} G_{f0}) \tag{S18}$$

where α_S is the geometrical scaling factor is related to the size of the crack shielding specimen, P_{SD} is the potential function of the shielded deflection derived by the angle compliance analysis. As expected, the theoretical model confirms the experimental results (Fig. 3c). This theoretical model explains the generally increasing trend of fracture energy over θ_s, and the additional fracture energy observed for the specimens with θ_s = 50º – 60º.

Supplementary Note 8. The angle compliance analysis for crack shielding

In this note, the detailed derivation and procedure of angle complaince analysis is given. Based on the experimental results (Fig. 3 and Supplementary Fig. 15) and simulation intepretations (Supplementary Fig. 10 and Supplementary Fig. 12). The shielded deflections events are observed for the shielding angles ranging from 50° to 60°. For the absolute differences of the positive and negative shielding angles with the absolute values close to 0° or 180°, the fracture will more likely to directly cut-through the shield units without deflections due to a limited resistance of the crack (close to 0°) or the highly disagreement of the deflection angles (close to 180°). In addition, higher fracture energies are also observed for the shield units with larger disagreement between the positive and negative shielding angles, which will contribute to the shielded deflections. To explain this coupled effect and model the fracture energy of different shield units, we model the probability of the shielded deflection by two terms with normalizations (Supplementary Fig. 30): (1) an exponentially increasing potential term reflecting the higher fracture energies with larger shielding angles, and (2) a gaussian distribution term reflecting the complaince of the shielding angles. The resulted formula is provided below:

$$P_{SD}(\theta_s) = A\exp(-\tau(\pi/2 - \theta_s))\exp(-(\theta_s - \mu_{\theta_s})^2/(2\sigma^2)) \quad \text{(S19)}$$

where $P_{SD}(\theta_s)$ is the potential function of the shielded deflection for the shielding angle θ_s, A is a normalization factor, τ, μ_{θ_s} and the σ are the fitting parameters, which is defined as τ = 0.4, μ_{θ_s}= 45, and σ = 5 in this work for the best fit of the experimental results.

Supplementary Note 9. Theoretical derivations of the fracture energy barriers for crack tip interactions

We applied FEA to model the average energy increment required for the crack tip to: (1) by-pass a CB phase, (2) cut-through a CB phase, and (3) propagate through a unit cell under the deflection of the CD phase with different separation distances d_{CD} (Fig. *3*e, Supplementary Fig. 16, Supplementary Fig. 17, and Supplementary Fig. 19a-b). G_{f0} is used to represent the fracture energy required to propagate through a base cell, Δ_a represents the fraction of energy increment for an event a compared to the energy of crack propagation without event a:

$$\Delta_a = (G_a - G_{f0})/G_{f0} \qquad \text{(S20)}$$

As the density of the CB phase increase, a larger possibility of the by-passing is expected, while the agglomeration of T1 cells is more frequently observed. An increased number of agglomerated crack bowing (CB) phases will lead to a larger potential of a crack cut-through events. In this note, the probabilities of the agglomerations are modelled with respect to different densities of the CB phases. For a propagating crack tip, the surface of the crack with potential encounter of agglomerated CB phases is described in Supplementary Fig. 31. Specifically, an agglomeration event would happen when three or more adjacent CB phases are observed along the direction vertical to the crack front, where one of those CB phases lies directly on the crack tip. Since the location of the crack tip is fixed, the probability for observing n agglomerations is:

$$P_{n\,CB2}(\rho_{CB}) = C_N^n {\rho_{CB}}^3 \qquad \text{(S21)}$$

where $P_{n\ CB2}(\rho_{CB})$ is the probability for n agglomerated CB phases with a density of ρ_{CB}, C_N^n is the mathematical combinations of choosing n indistinguishable items with a total number of items N. To model the statistical energy increment of the cut-through of CB phases, we scaled the probability by the number of the agglomerations by:

$$P_{CB2}(\rho_{CB}) = \sum_{n=1}^{N} n\, C_N^n \rho_{CB}{}^3 \tag{S22}$$

Therefore, we model the $G_{f,CB}$ as

$$G_{f,CB} = \alpha_{CB}\rho_{CB}[\Delta_{CB1}G_{f0} + \Delta_{CB2}P_{CB2}G_{f0}] \tag{S23}$$

where the α_{CB} is the geometrical scaling factor related to the size of the crack bowing specimen, ρ_{CB} is the density of the CB phase, P_{CB2} is the scaled possibility of the cut-through event derived by the statistical modeling of agglomeration events. For the crack deflected by the CD phases, we observed smaller Δ_{CD} for loosely-distributed CD phases with larger d_{CD}, indicating that the deflection is less effective per unit cell propagation for increasing d_{CD}. Based on these analysis, we can hence model the additional energy $G_{f,CD}$ as:

$$G_{f,CD} = \alpha_{CD}\Delta_{CD}l_f G_{f0} \tag{S24}$$

where the α_{CD} is the geometrical scaling factor related to the size of the crack deflection specimen, l_f is the actual length of the fracture path. As expected by experimentally fitting equations (S23-S24) for α_{CB} and α_{CD}, the proposed theoretical model well-described the fracture energy of the experimental results with pure crack bowing and deflection events (Fig. 3a-b).

Supplementary Note 10. Theoretical derivations of the fracture energy barriers for reinforcement bridging

For the reinforcement bridging, we further model the fracture energy of the metamaterials with reinforcement bridges as:

$$G_{f,RB} = \alpha_{B1}\Delta_{CD}O_{BD}G_{f0} + \alpha_{B2}\Delta_{CB2}O_{BC}G_{f0} + \alpha_{B3}\Delta_{B}\mathrm{A}_{B}S_{B}G_{f0} \qquad \text{(S25)}$$

where $G_{f,B}$ is the fracture energy of the metamaterials with crack bridging, α_{B1}, α_{B2}, and α_{B3} are the geometrical scaling factors for the additional fracture energies caused by bridged deflection, bridged cut-through, and bridging, respectively, Δ_B is the energy increment per bridging surface produced by pure bridging, O_{BD} and O_{BC} represents the occurrences of the bridged deflection and bridged cut-through, respectively. Since the fracture behaviors of the bridged cut-through and deflection are similar to the phenomena of crack tip interactions, we directly used the previously calculated energy increments of the crack cut-through and crack deflections, Δ_{CB2} and Δ_{CD}. To reveal the energy increment of the bridging, we simulated the fracture process at the crack tip with different sizes of the bridging fibers (Fig. 3e, Supplementary Fig. 18, and Supplementary Fig. 19d). The simulations indicate that a significant amount of stress at the crack tip is shared by the bridging fibers, which produces a 3% - 5% improvements of the fracture energy per area of the bridging surfaces compared to the one without crack bridging. Based on these theoretical derivations, we calculated the fracture energy of each specimen and compared it with the experimental results (Fig. 3d). According to the experimentally recorded bridging events (Supplementary Fig. 15b), the theoretical model predicts that the fracture energies of the bridging specimens with S_B = 3 exhibit an obvious growth over larger d_B

due to an increased A_B, where the energy increments contributed by the bridging dominates. For the specimens with larger sizes of bridging fibers, the A_B does not strictly increases over d_B, and the combining effects of the occurrences for the bridging, bridged deflection, and bridged cut-through causes a more significant energy improvements for d_B = 3 cells and d_B = 5 cells.

Supplementary Note 11. Mechanisms of fracture toughening events

We engineer specimens with four types of micro-scale toughening units: (1) crack front bowing specimens, where 10% to 50% conventional base cells of the specimen is random1y selected and converted to CB phases constructed by T1 DP cells that impede the crack motion, (2) crack deflection specimens, which are uniformly embedded with 3 × 3 × 3 secondary CD phases composed of both T1 and T2 DP cells, (3) specimens with shielding units, which are engineered with T3 DP cells that enables the crack blunting, and (4) specimens with reinforcement bridges, in which arrays of T1 cells are used to construct periodic reinforcement fibers with different lengths and bridging distances. It is observed that the crack is forced to by-pass and cut-through the CB phases (Supplementary Fig. 13) to overcome additional energy barriers $\Delta_{CB1}G_{f0}$ and $\Delta_{CB2}G_{f0}$, respectively (Supplementary Fig. 8). The deflected crack from CD phase exhibited increased actual length of the crack path (Supplementary Fig. 14), where the blunted crack tip and extended crack path provide additional fracture energy as $\Delta_{CD}l_f$ (Supplementary Fig. 9). Affected by the angles of shield units (θ_s), a shielded crack tip displayed a well-distributed and less-concentrated stress field and potential and shielded deflections (Supplementary Fig. 10 and Supplementary Fig. 15), leads to added fracture energy as $\Delta_S G_{f0} + P_{SD}\Delta_{SD}G_{f0}$. The implanted bridge with different bridge distance (d_B) brings different energy events including the crack delfection, cut-through, and bridged crack fronts (Supplementary Fig. 16 and Supplementary Fig. 11), which can be described with $\Delta_{CD}O_{BD}G_{f0}$, $\Delta_{CB2}O_{BC}G_{f0}$, and $\Delta_B \mathrm{A}_B S_B G_{f0}$.

CB specimens exhibited both by-pass and cut-through of CB phases, where an increasing number of bowing events, extended fracture plateau, and additional fracture

energies are experimentally observed (Supplementary Fig. 13) for specimens with a higher density of CB phases (ρ_{CB}). Simulation results (Supplementary Fig. 8) supported the experimental observation that overcoming energy barriers, $\Delta_{CB1}G_{f0}$ and $\Delta_{CB2}G_{f0}$, was necessary for by-passing isolated CB phases or cut-through of agglomerated CB phases, respectively. Crack deflection specimens exhibited increased actual length of the crack path due to complete deflection of crack tips by CD phases, where more deflection events and resulted fracture energies were experimentally observed (Supplementary Fig. 14) for specimens with denser CD phase. This is further confirmed by the numerical results (Supplementary Fig. 9) that the out-of-plane deflection blunts the crack tip, extends of the actual crack path, and provides additional fracture energy as $\Delta_{CD}l_f$. In addition, experimental results (Supplementary Fig. 15) suggested that specimens with uniform shield units of different shield angles (θ_s) exhibits the most significant improvement of fracture energies for 50º - 60º shielding angles. Numerical simulations (Supplementary Fig. 10) further revealed that a shielded crack tip displayed a well-distributed stress field with reduced stress concentration, effectively blunting the crack and introducing additional shielding energy for crack propagation. The angle compliance analysis (Supplementary Note 6) established the theoretical correlation between the occurrences of shielded deflections and θ_s, providing an explanation for the maximum energy absorption observed at 50º - 60º shielding angles. The study of reinforcement bridging involved experimental measurement of fracture energy in specimens with varying bridging parameters (Supplementary Fig. 16), revealing that reducing the bridge distance (d_B) enhances crack deflection, whereas increasing d_B promotes cut-through of bridging fibers. The energy increments of experimentally observed bridged cut-through, deflection, and crack-bridging

events were simulated using fracture processes at the crack tip with varying sizes of the bridging fibers (Supplementary Fig. 11), thereby completing the theory in the main text.

Supplementary figures and tables

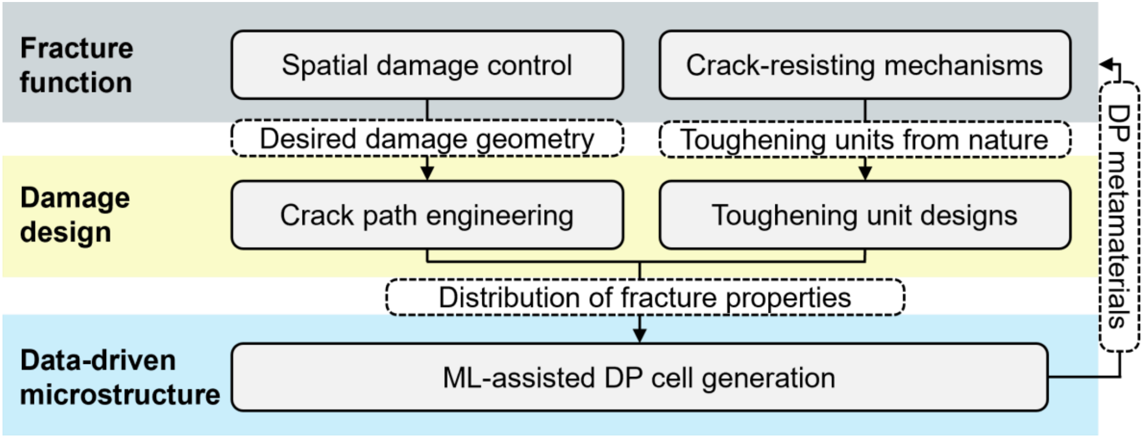

Supplementary Fig. 1. The schematics of data-driven damage-programming design, where the term DP represents damage-programmable.

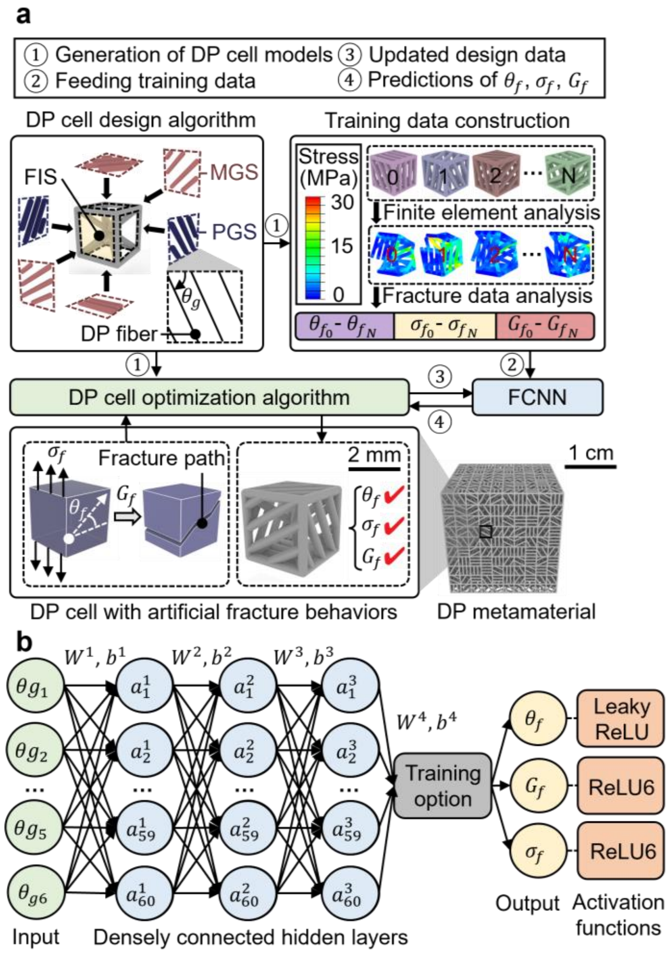

Supplementary Fig. 2. Data-driven design of damage-programmable (DP) cells. (a) The overall design flowchart, where θ_g is the guiding angle, θ_f is the fracture angle, G_f represents the fracture energy, σ_f is the fracture strength, FIS, PGS, MGS are the abbreviations for fracture initiation surface, primary guiding surface, and minor guiding surface, respectively. (b) The structure and hyperparameters of the fully connected neural networks (FCNNs), where, a_i^1 to a_i^3 are the output values of the ith neuron in layer 1 to layer 3, W^i and b^i are the weight matrix and bias vector between layer i and $i-1$, respectively, the number of neurons is represented by n, which is selected as 30, 40, and 50 to predict θ_f, σ_f, and and G_f, respectively, ReLU is the rectified linear unit.

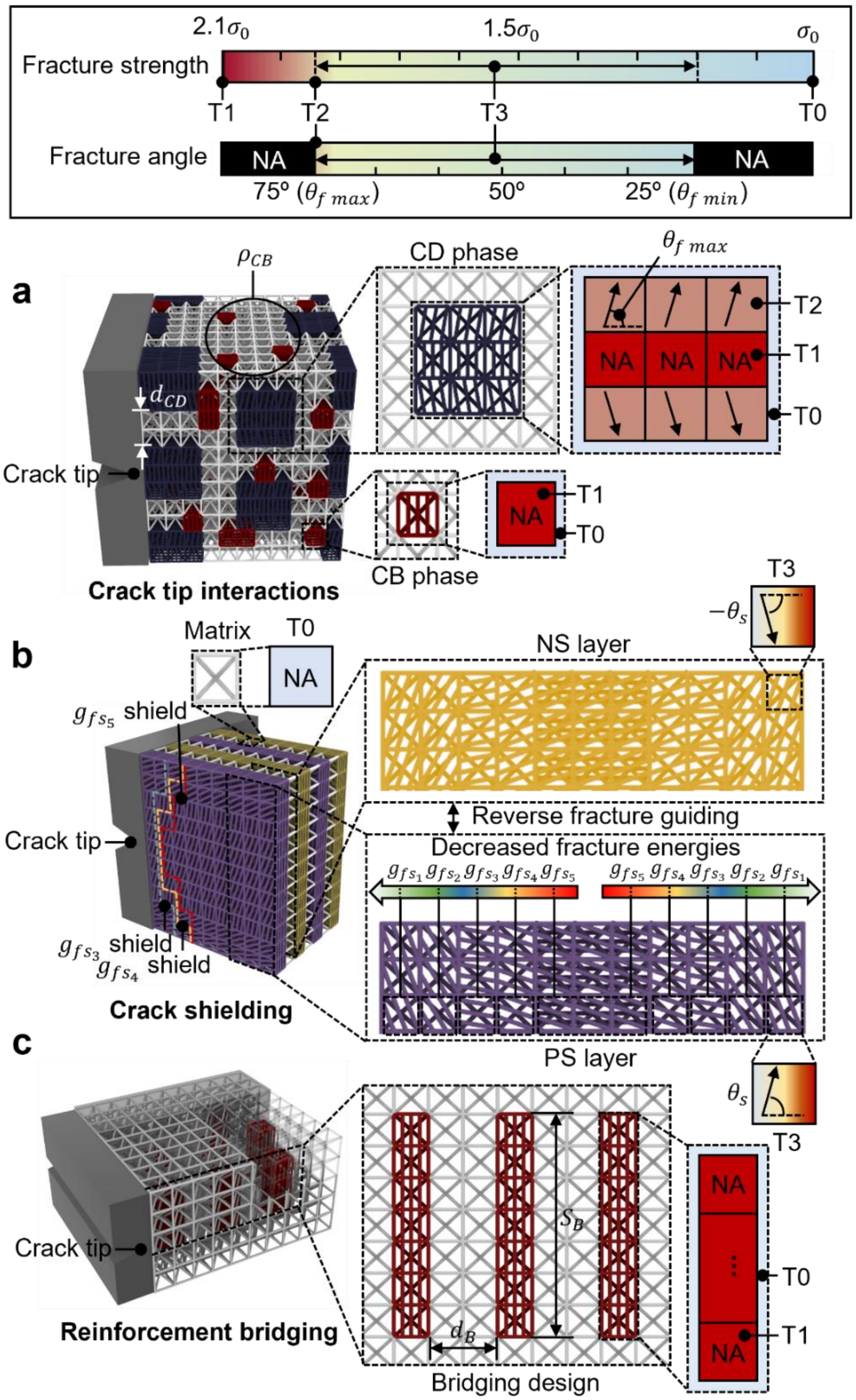

Supplementary Fig. 3. Achieving the fracture-resisting mechanisms in nature by damage-programmable (DP) metamaterials. (a) The DP metamaterials with crack tip interactions,

where d_{CD} is the distance between the adjacent crack deflection (CD) phases, T0 represents the base cells, T1 is the type of DP cells with highest fracture strength with zero fracture angles, T2 is the type of DP cells that has the largest fracture strength for the maximum fracture angles $\theta_{f\ max}$, ρ_{CB} is the density of crack bowing (CB) phase. Note that σ_0 is the fracture strength of T0 cells. (b) The DP metamaterials with crack shielding, θ_s is the guiding angle of the shield units, T3 is the type of DP cells with engineerable fracture angle of θ_s ranging from minimum to maximum achievable DP fracture angles $\theta_{f\ min}$ to $\theta_{f\ max}$, g_{f_1} - g_{f_5} are the fracture energies of different shield units. NS and PS represents the negative and positive shielding layers of the shield units, respectively. (c) The DP metamaterials with reinforcement bridging, where S_B is the size of the bridging fiber, d_B is the distance between adjacent bridging fibers.

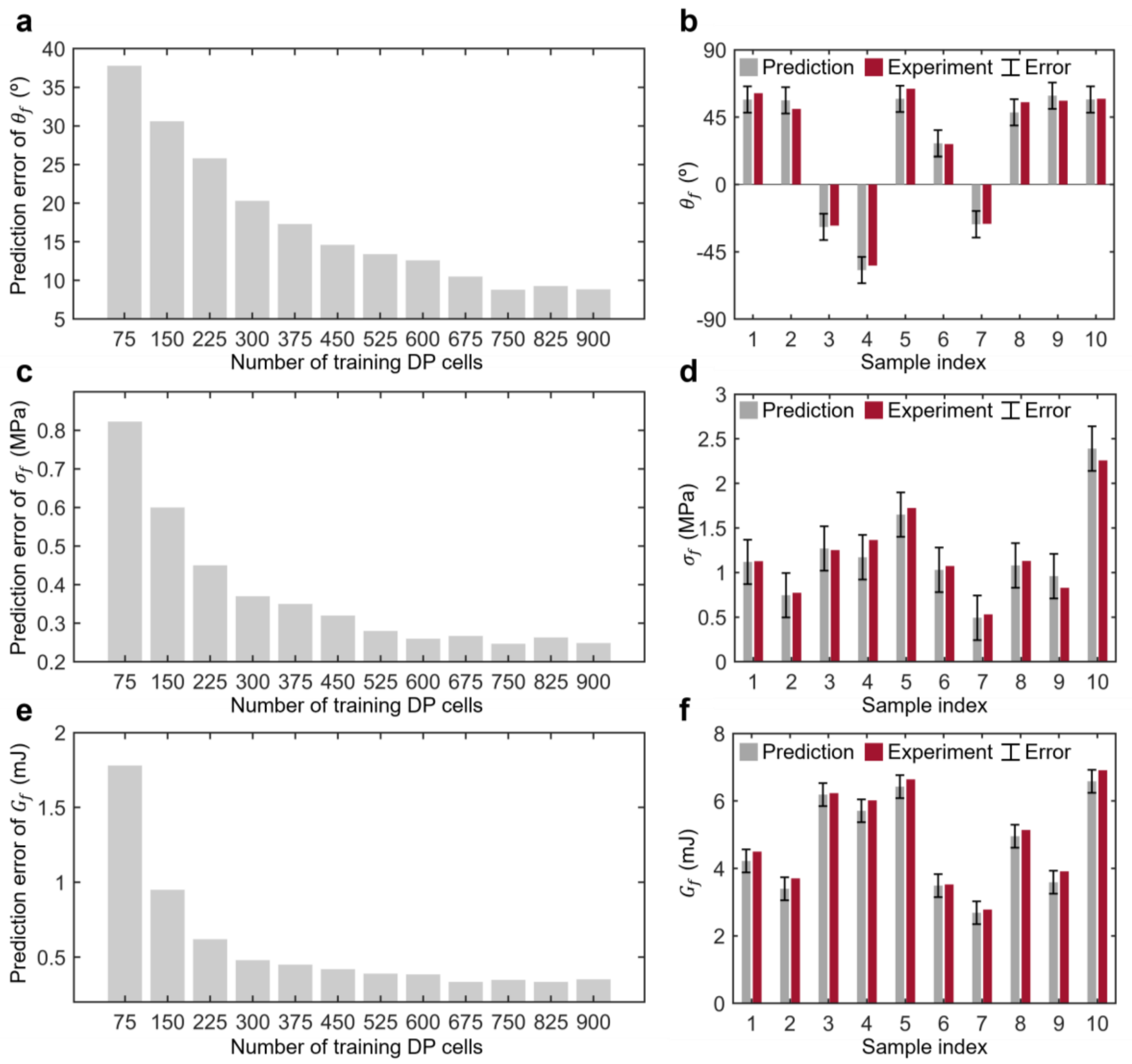

Supplementary Fig. 4. Prediction errors and experimental validations of the fully connected neural networks (FCNNs). (a-b), (c-d), (e-f) provides the prediction errors and experimental validation results of the fracture angle (θ_f), fracture strength (σ_f), and fracture energy (G_f) for damage-programmable (DP) cells, respectively. The error bars indicate the prediction error of the FCNNs. Source data are provided as a Source Data file.

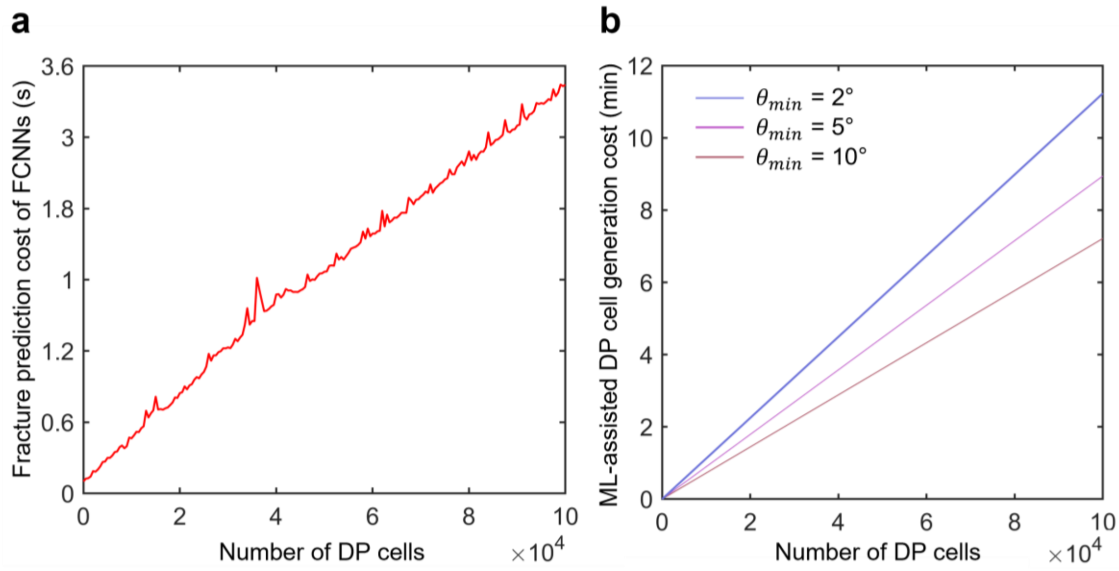

Supplementary Fig. 5. Computational cost analysis. (a) Fracture prediction using fully connected neural networks (FCNNs). (b) Machine-learning-assisted (ML-assisted) damage-programmable (DP) cell generation featuring deep search refinement coefficient β = 0.1, with 1000 DP cells randomly generated per search cycle and angle resolution θ_{min} = 2-10º. Source data are provided as a Source Data file.

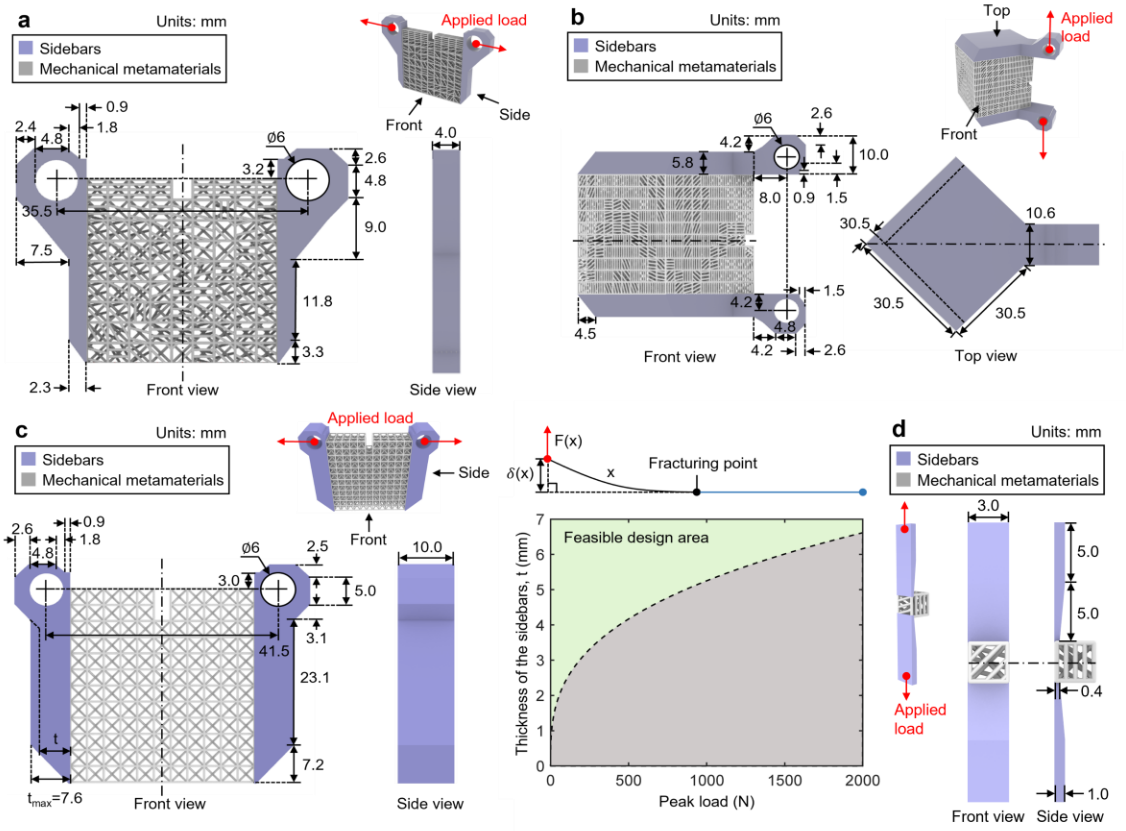

Supplementary Fig. 6. Specimen designs. (a-c) Designs of the fracture specimens for the evaluation of 2D crack propagation, evaluation of 3D crack propagation, and the measurement of fracture energies, respectively, where the feasible design area of the sidebars is determined by a simplified bending model. (d) Designs of specimens for the single damage-programmable (DP) cell fracture measurement. Source data are provided as a Source Data file.

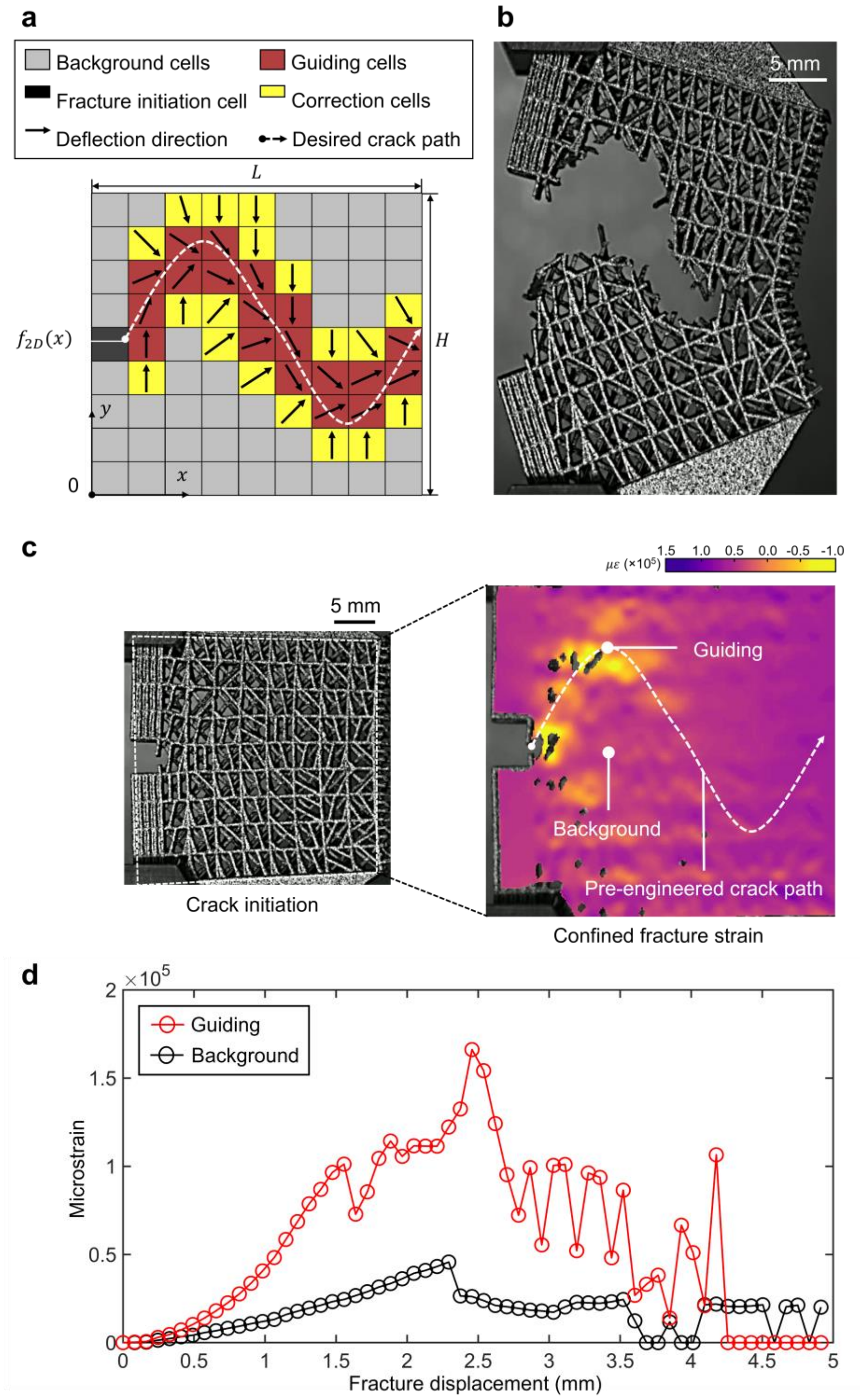

Supplementary Fig. 7. Data-driven fracture path engineering using the damage-programmable (DP) metamaterials for a 2D sine-shaped crack path. (a) The digital design of crack path described by $f_{2D}(x) = (H/3)sin[(2\pi/L)x] + H/2$, where $f_{2D}(x)$ is the

function describing the 2D crack geometry, L and H is the length and height of the 2D design space, respectively. (b) Experimentally measured actual 2D fracture path. (c) The digital image correlation (DIC) analysis of the controlled 2D fracture propagation, where $\mu\varepsilon$ represents the microstrain. (d) The strains at the guiding and background regions for the DP metamaterials based on the digital design of pre-engineered fractures. Source data are provided as a Source Data file.

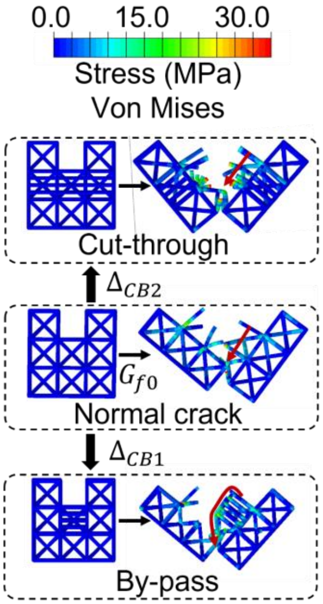

Supplementary Fig. 8. Simulated fracture process of the normal propagation, by-pass, and cut-through events. Δ_{CB1} and Δ_{CB2} represents the energy increment fractions for the by-pass and cut-through crack bowing events, respectively.

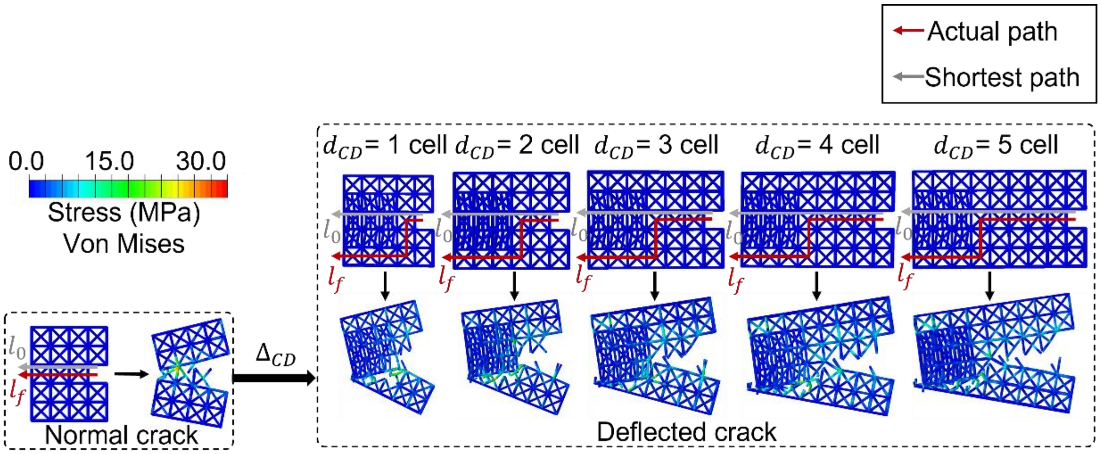

Supplementary Fig. 9. Simulated fracture process of the normal propagation and crack deflection events with different distance between the crack deflection (CD) phases, d_{CD}. Δ_{CD} represents the energy increment fraction for the crack deflection events.

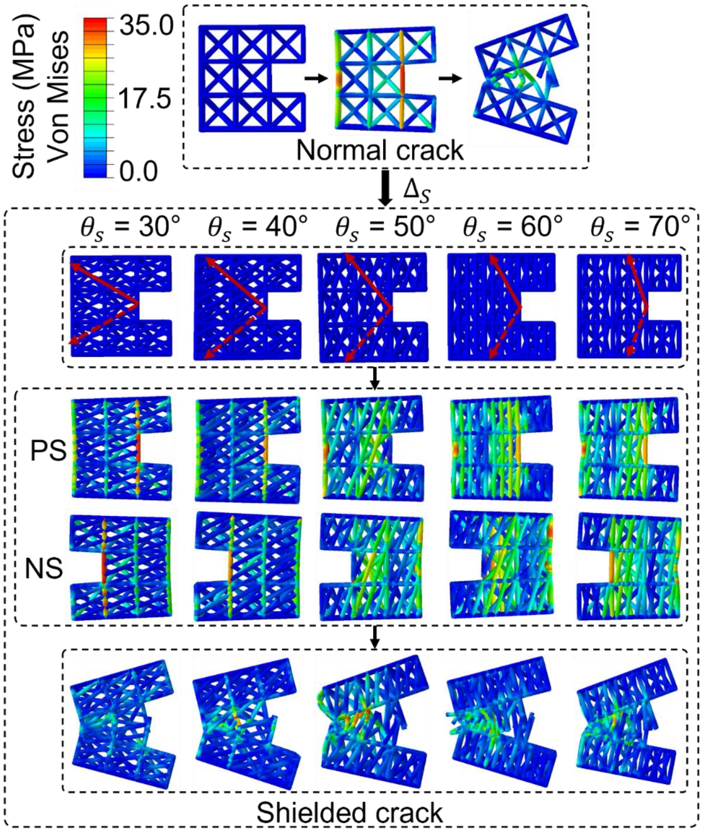

Supplementary Fig. 10. Simulated fracture process of the normal propagation, and the crack shielding with shield angle $\theta_s = 30° - 50°$. Δ_S represents the energy increment fraction for the shieled crack events. NS and PS are the layers with negative and positive shielding.

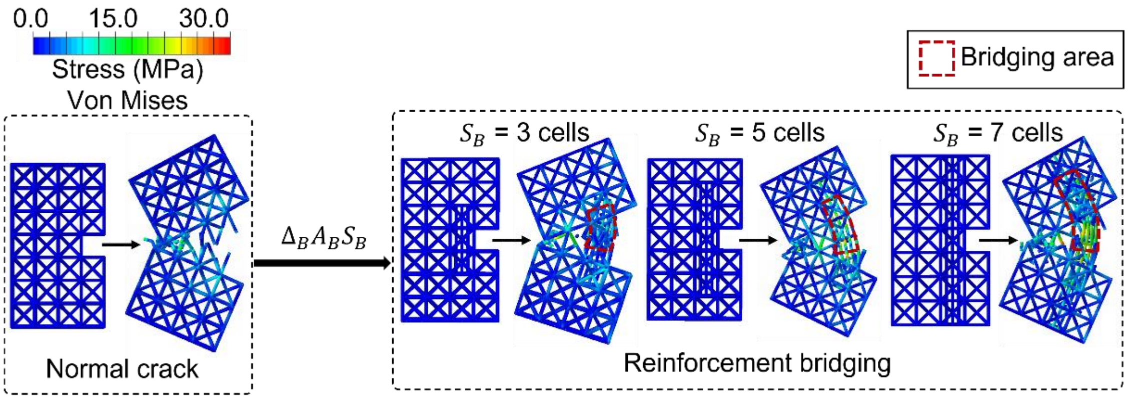

Supplementary Fig. 11. Simulated fracture process of the normal propagation, and the reinforcement bridging with sizes of the bridge S_B ranging from 3 to 7 cells. Δ_B represents the energy increment fraction for the bridged crack events. A_B is the statistical bridging area per length of the bridging fibers.

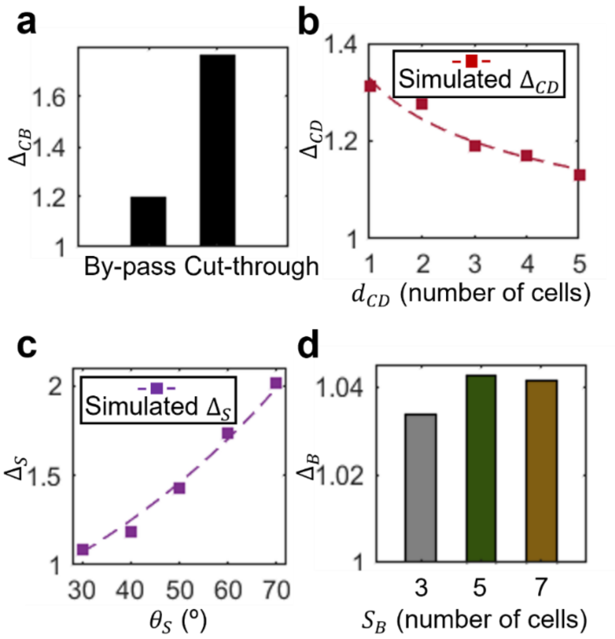

Supplementary Fig. 12. The numerically modelled fracture energy increment of different fracture events. (a) Crack bowing. (b) Crack deflection. (c) Shield units. (d) Reinforcement bridging. Δ_{CB}, Δ_{CD}, Δ_S, and Δ_B are energy increment fractions for crack bowing, crack deflection, shielded crack, and bridged crack events, respectively. d_{CD}, θ_S, and S_B are the distance between crack deflection phases, shield angle, size of the bridges, respectively. Source data are provided as a Source Data file.

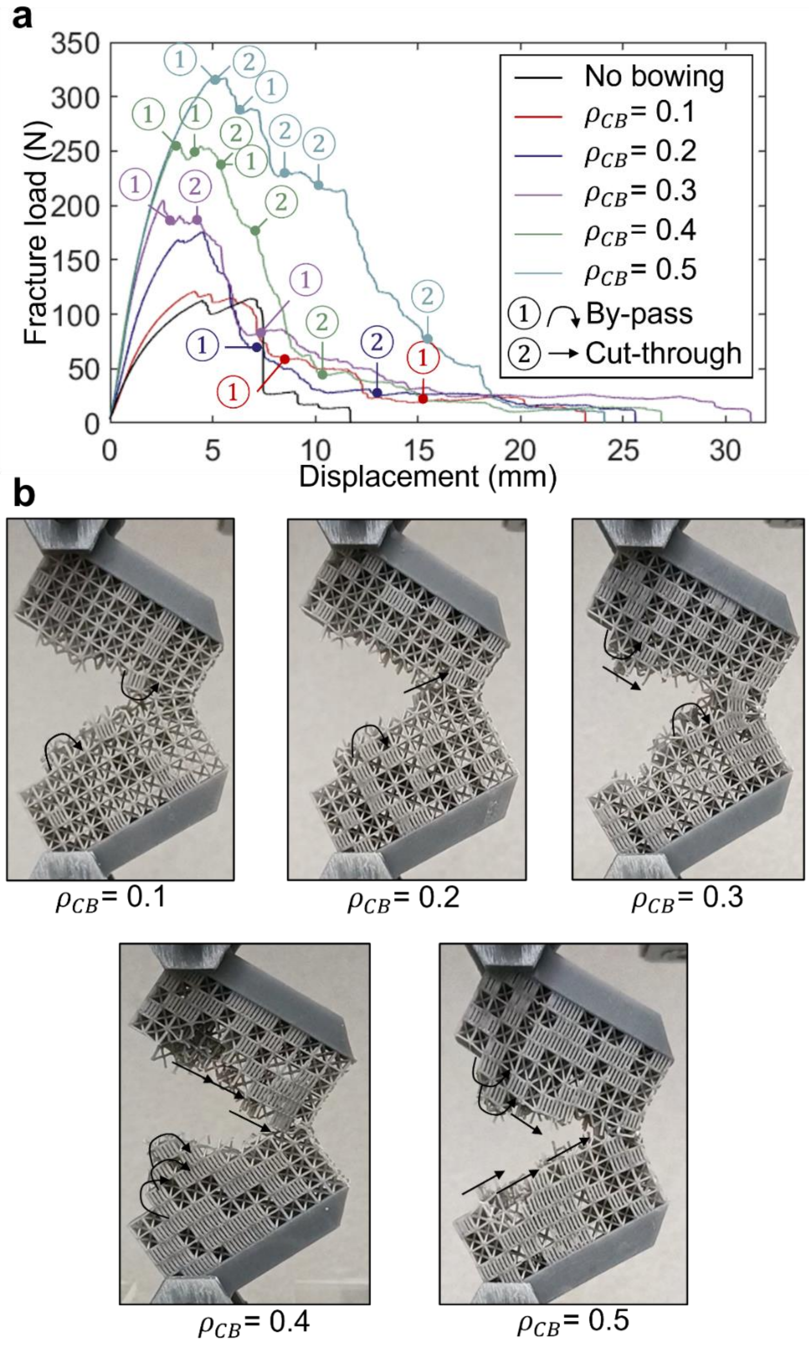

Supplementary Fig. 13. Fracture responses and experimental captures of crack bowing specimens. (a) Fracture load displacement curves and (b) snapshots of the fracture processes for the crack bowing specimens with 10% – 50% randomly added CB phase, where ρ_{CB} is the density of CB phase. Source data are provided as a Source Data file.

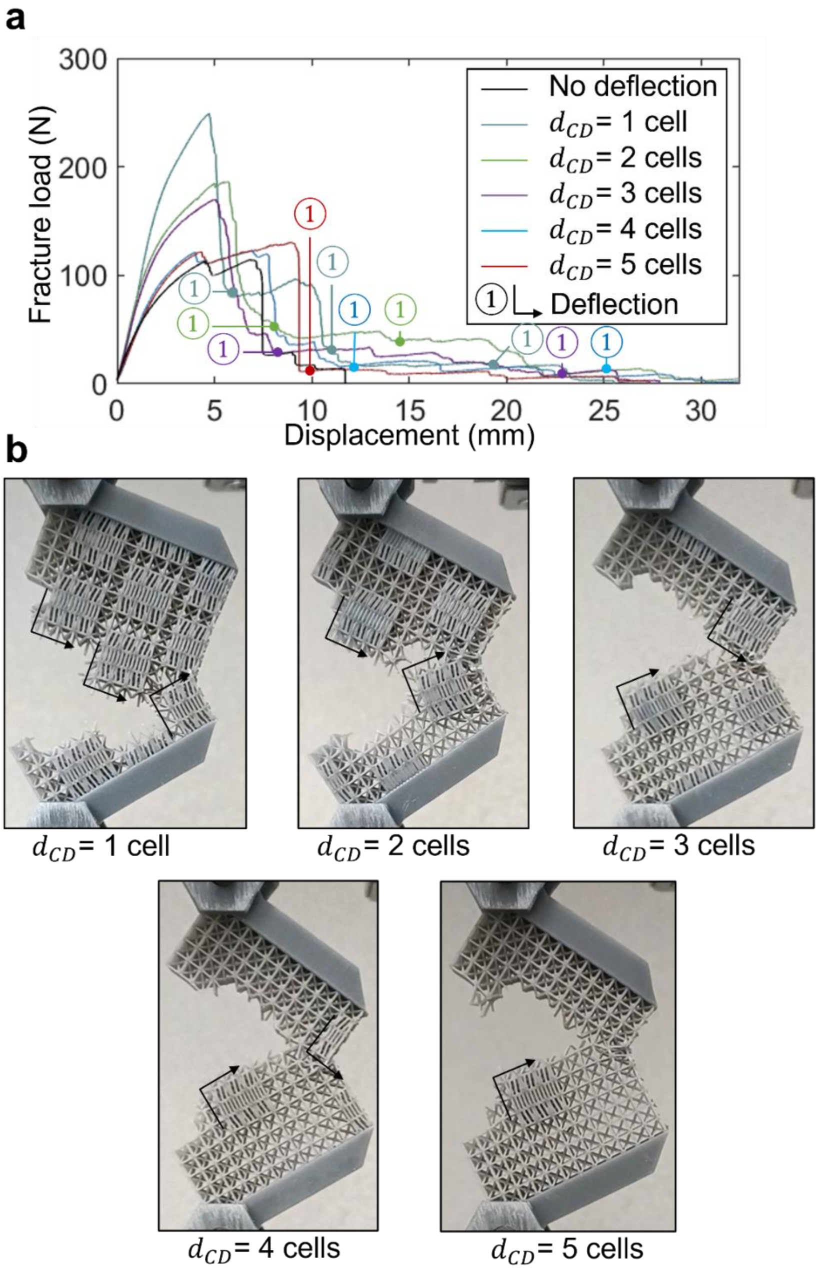

Supplementary Fig. 14. Fracture responses and experimental captures of crack deflection specimens. (a) Fracture load-displacement curves and (b) snapshots of the fracture processes for the crack deflection specimens with uniformly distributed CD phase separated by 1 – 5 cells, where d_{CD} is the distance between the adjacent CD phases. Source data are provided as a Source Data file.

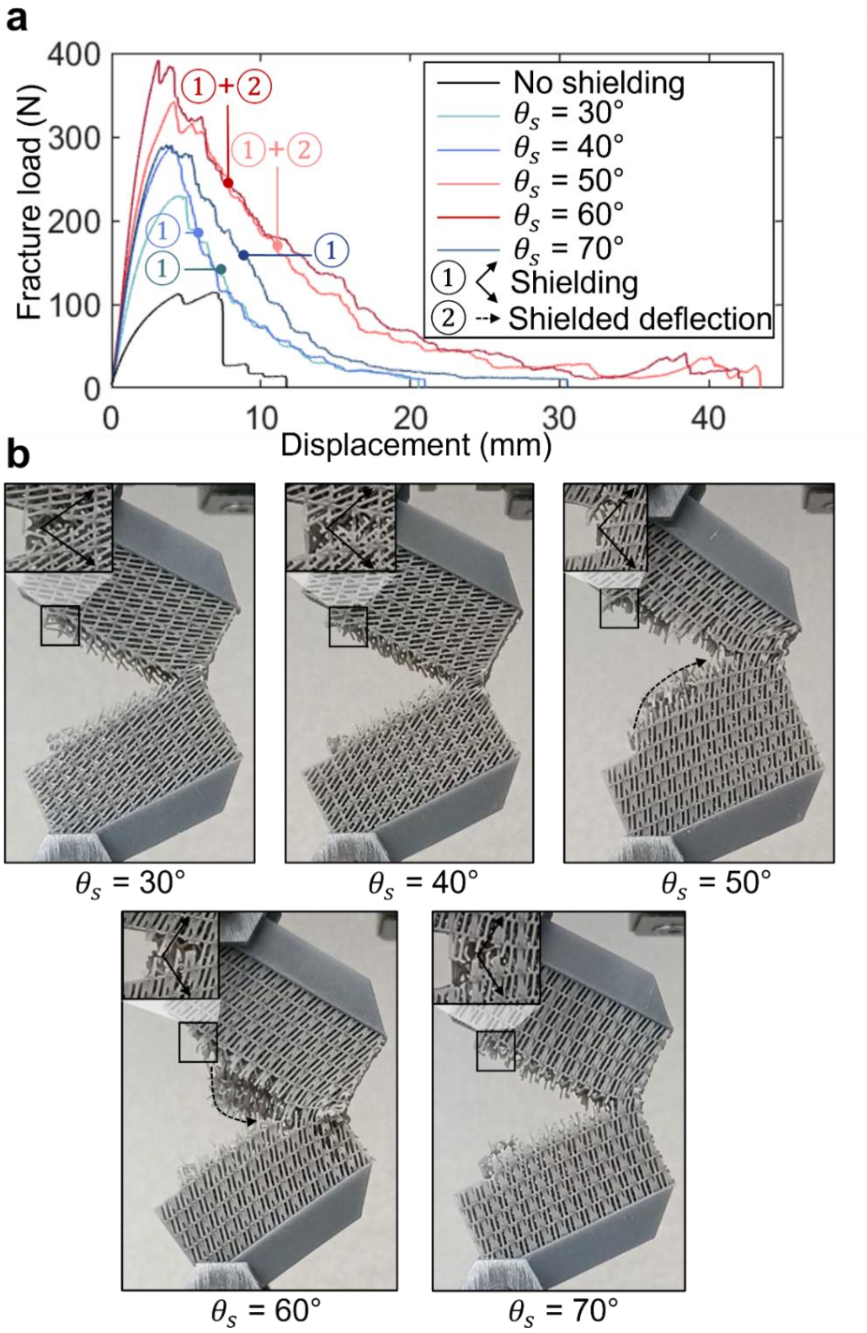

Supplementary Fig. 15. Fracture responses and experimental captures of specimens with shield units. (a) Fracture load displacement curves and (b) snapshots of the fracture processes for the specimens consist of uniform shield units with $\theta_s =$ 30° – 50°, where θ_s is the shielding angle. Source data are provided as a Source Data file.

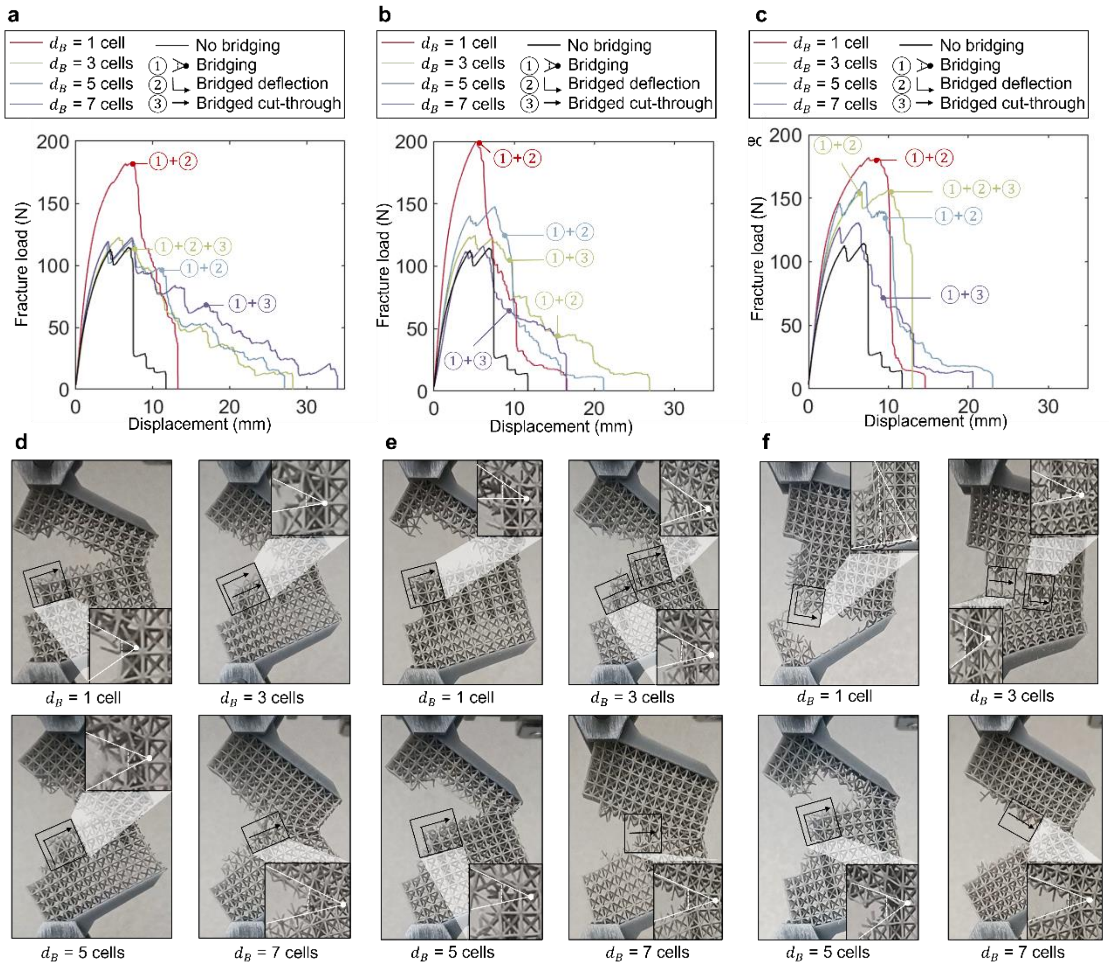

Supplementary Fig. 16. Fracture responses and experimental captures of crack bridging specimens. (a-c) Fracture load displacement curves and (b-d) snapshots of the fracture processes for the crack bridging specimens with size of the bridge S_B = 3 cells, S_B = 5 cells, and S_B = 7 cells, respectively. d_B is the distance between the bridges. Source data are provided as a Source Data file.

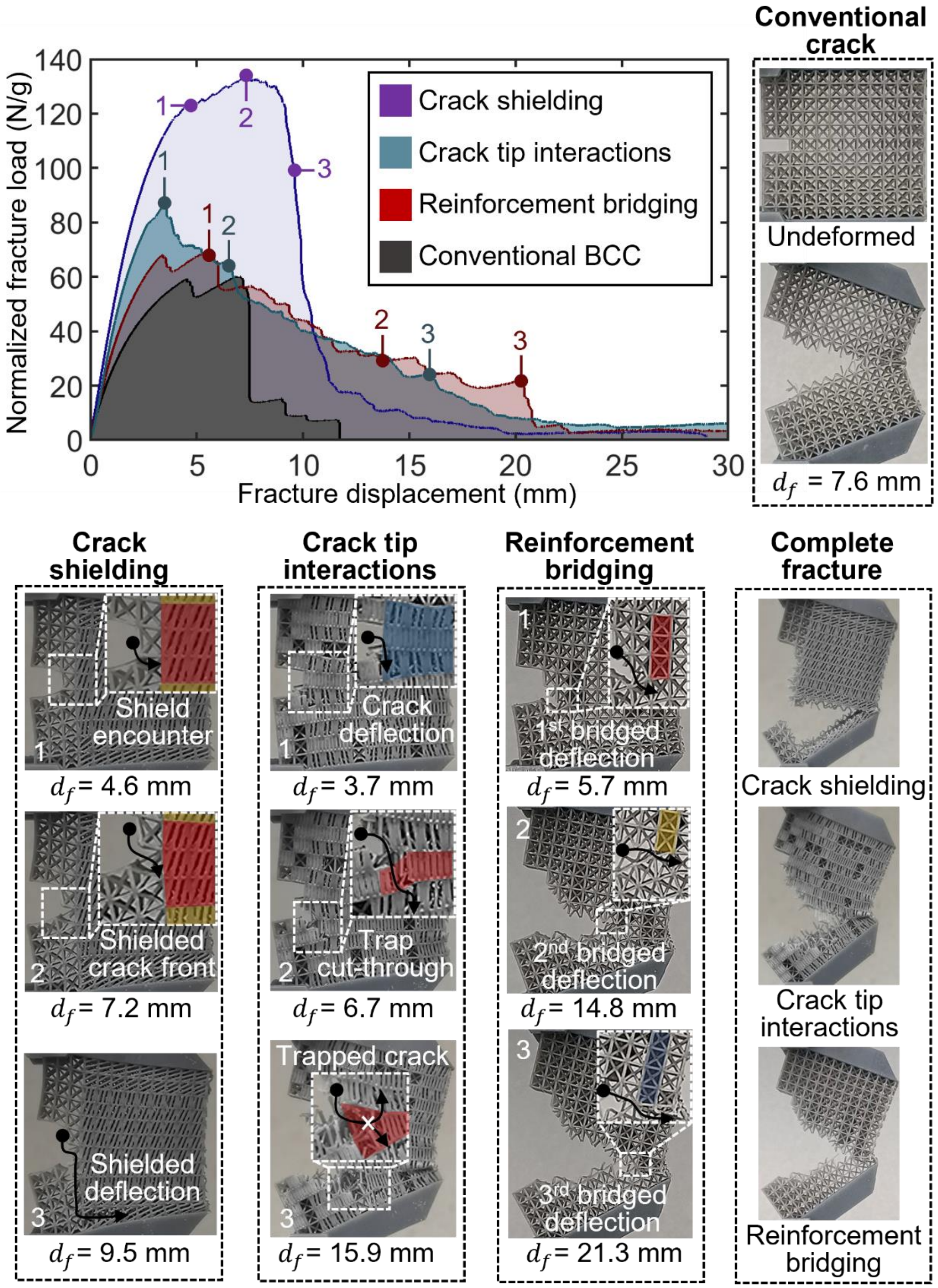

Supplementary Fig. 17. Normalized experimental fracture load displacement curves for the conventional body-centered-cubic (BCC) specimen and damage-programmable (DP) metamaterials with crack tip interactions, crack shielding, and reinforcement bridging. d_f is the fracture displacement. Source data are provided as a Source Data file.

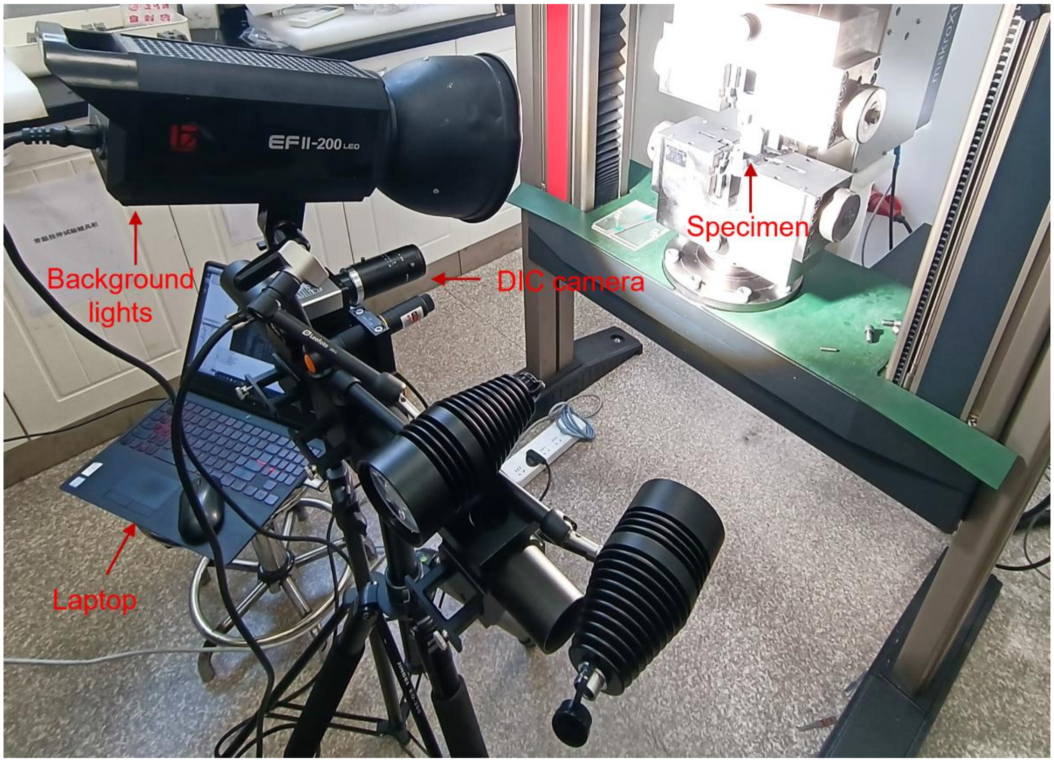

Supplementary Fig. 18. Experimental configuration of the digital image correlation (DIC) analysis.

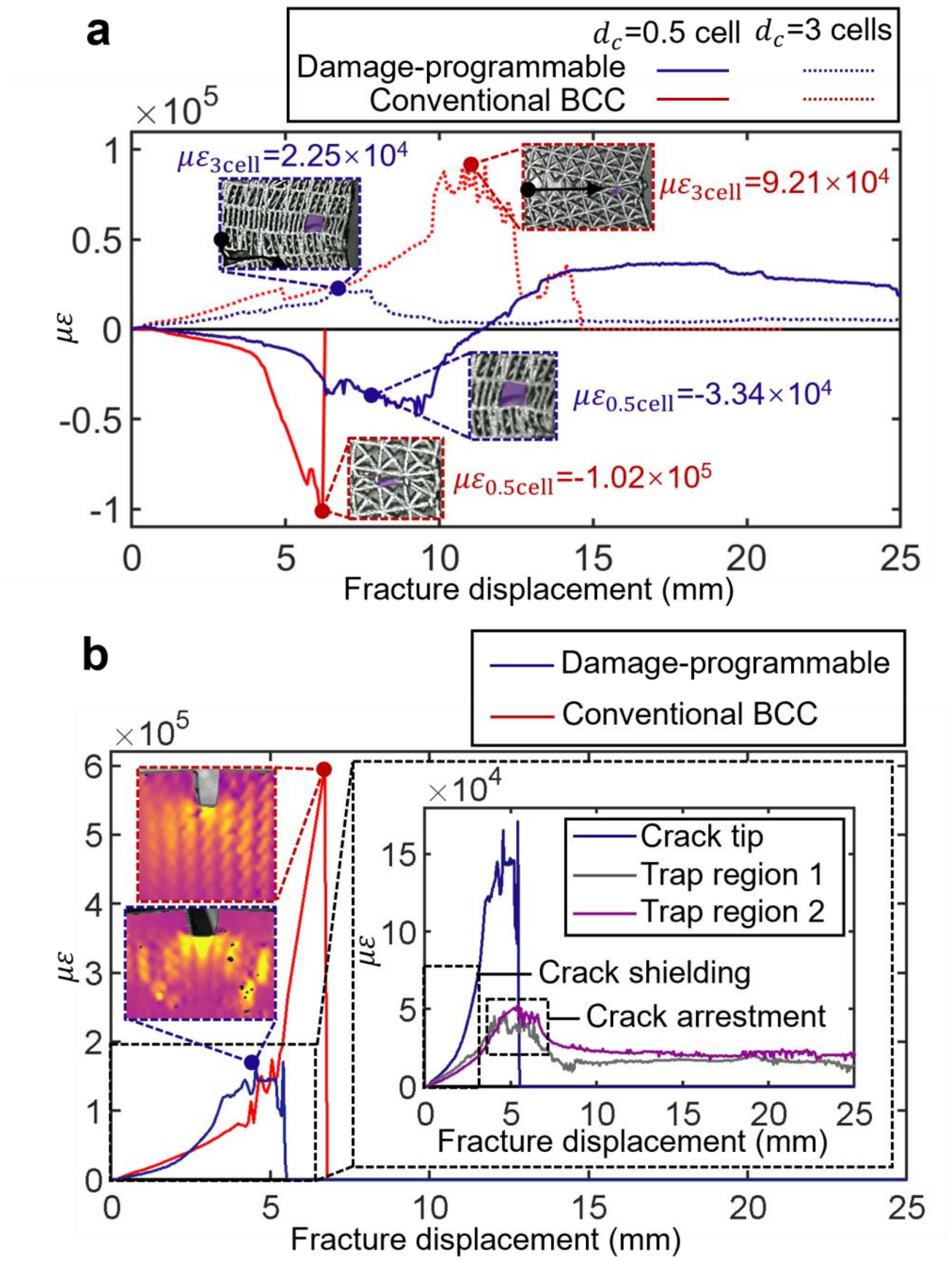

Supplementary Fig. 19. The strain data of the damage-programmable (DP) metamaterials with combined crack resistances and conventional body-centered-cubic (BCC) specimens. (a) Strain data to quantify crack-resisting events. (b) Strain data to quantify protection of the critical unit. d_c is the distance to the crack front, $\mu\varepsilon$ is the microstrain. Source data are provided as a Source Data file.

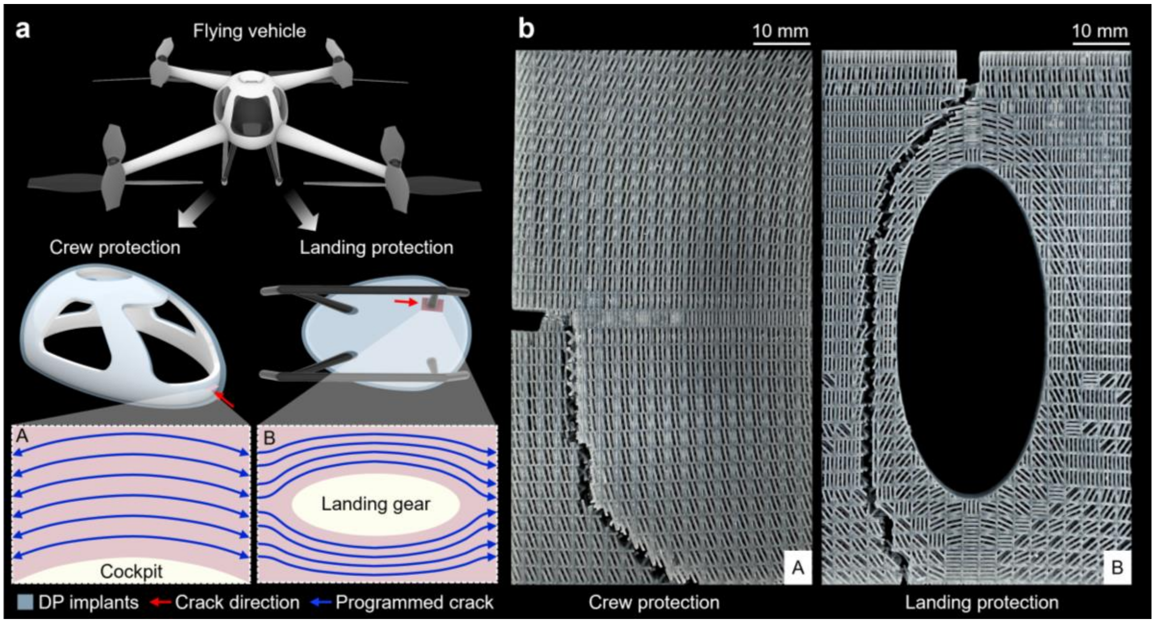

Supplementary Fig. 20. Demonstration of potential applications of damage-programmable (DP) metamaterials. (a) A flying vehicle enhanced with lightweight DP implants designed to guide cracks away from critical areas for improved safety. (b) Experimental validation of representative sections of DP implants.

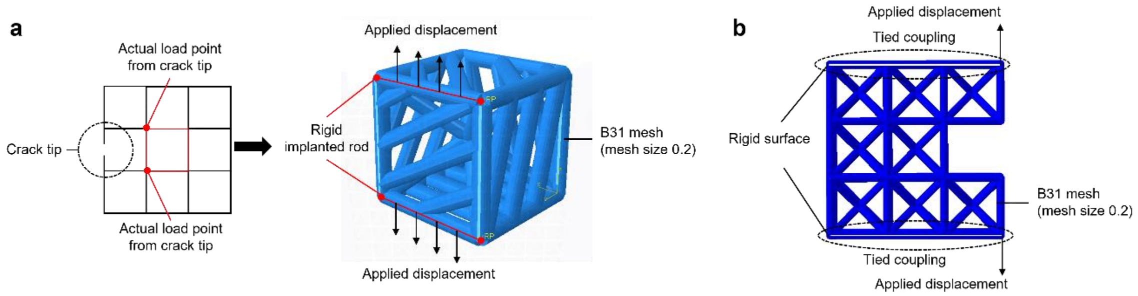

Supplementary Fig. 21. The simulation configurations. (a) Simulation configuration for the training data generation. (b) Simulation configuration for the local fracture process for different toughening mechanisms.

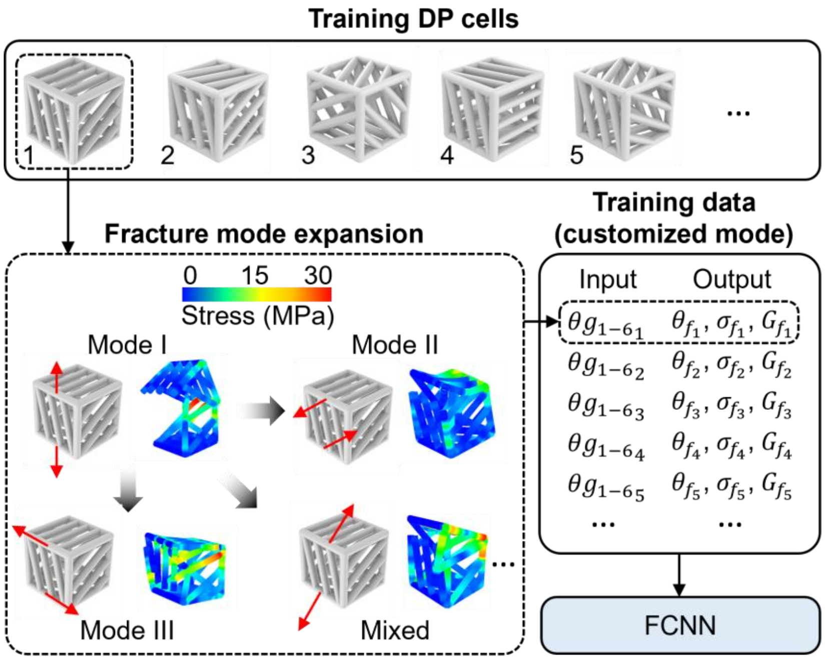

Supplementary Fig. 22. Extension of the predictive scope of fully connected neural networks (FCNNs) utilizing training data from arbitrary fracture modes.

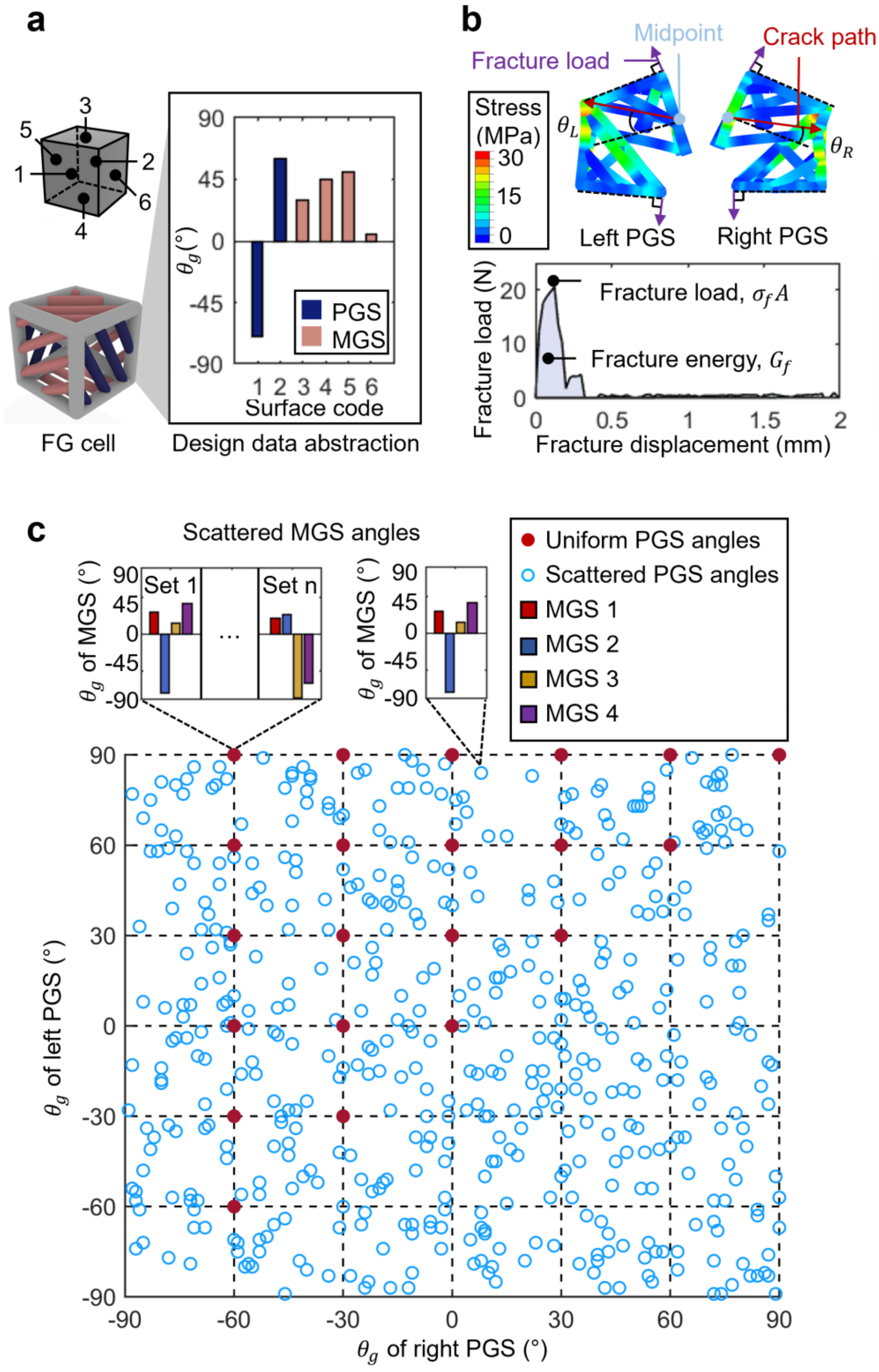

Supplementary Fig. 23. The construction of training data. (a) The design data abstraction of damage-programmable (DP) cells, where PGS and MGS represent the primary and

minor guiding surfaces, respectively. (b) The derivation of the training properties for a sample training DP cell, where θ_L and θ_R are the fracture angles of the left and right PGS, respectively, θ_g is the fracture guiding angle, θ_f is the fracture angle of DP cell, G_f represents the fracture energy, σ_f is the fracture strength. (c) The distribution of the training angles. Source data are provided as a Source Data file.

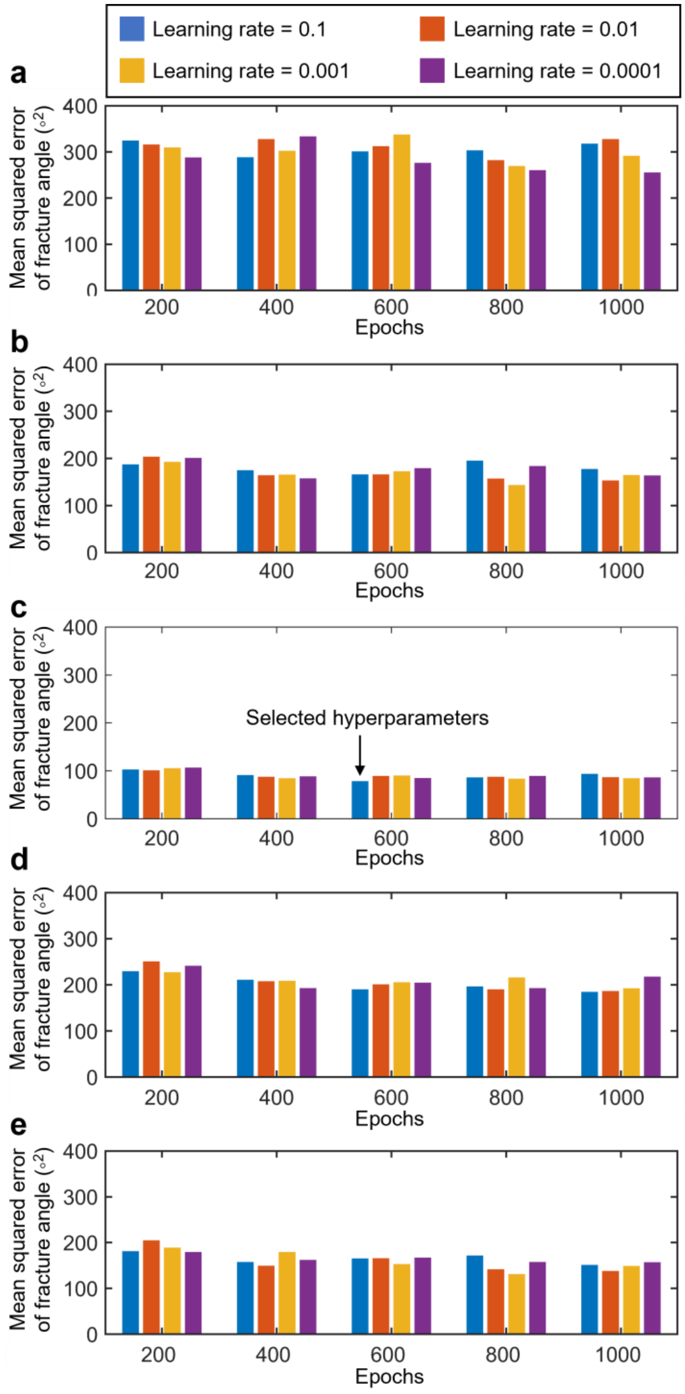

Supplementary Fig. 24. The average losses of fully connected neural network (FCNN) with different compositions of hyperparameters for fracture angle predictions. (a-e) The average losses for the FCNNs constructed with 10, 20, 30, 40, and 50 number of neurons in each layer, respectively. Source data are provided as a Source Data file.

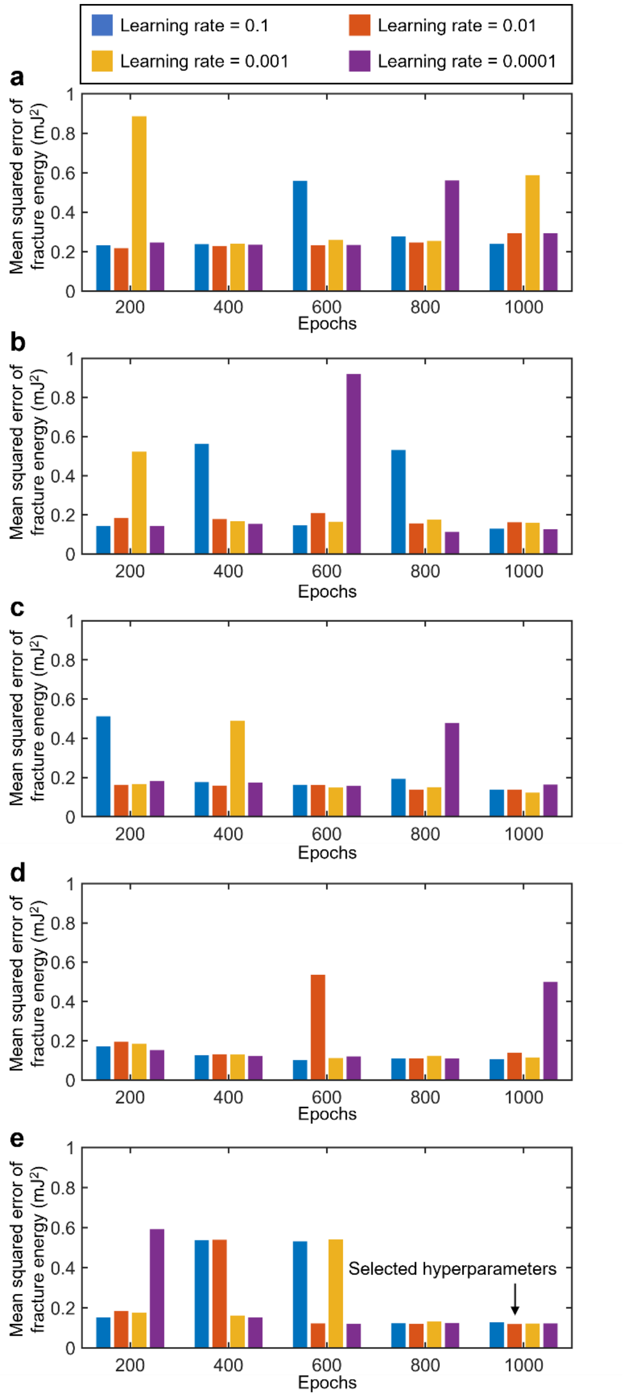

Supplementary Fig. 25. The average losses of fully connected neural network (FCNN) with different compositions of hyperparameters for fracture energy predictions. (a-e) The average losses for the FCNNs constructed with 10, 20, 30, 40, and 50 number of neurons in each layer, respectively. Source data are provided as a Source Data file.

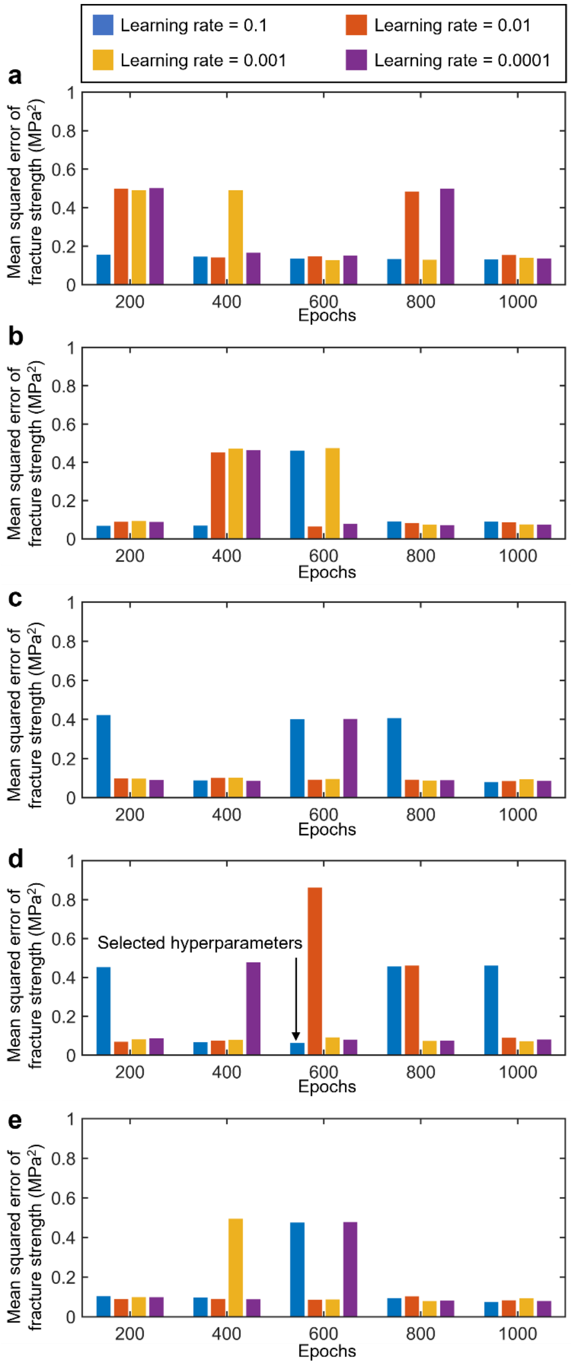

Supplementary Fig. 26. The average losses of fully connected neural network (FCNN) with different compositions of hyperparameters for fracture strength predictions. (a-e) The average losses for the FCNNs constructed with 10, 20, 30, 40, and 50 number of neurons in each layer, respectively. Source data are provided as a Source Data file.

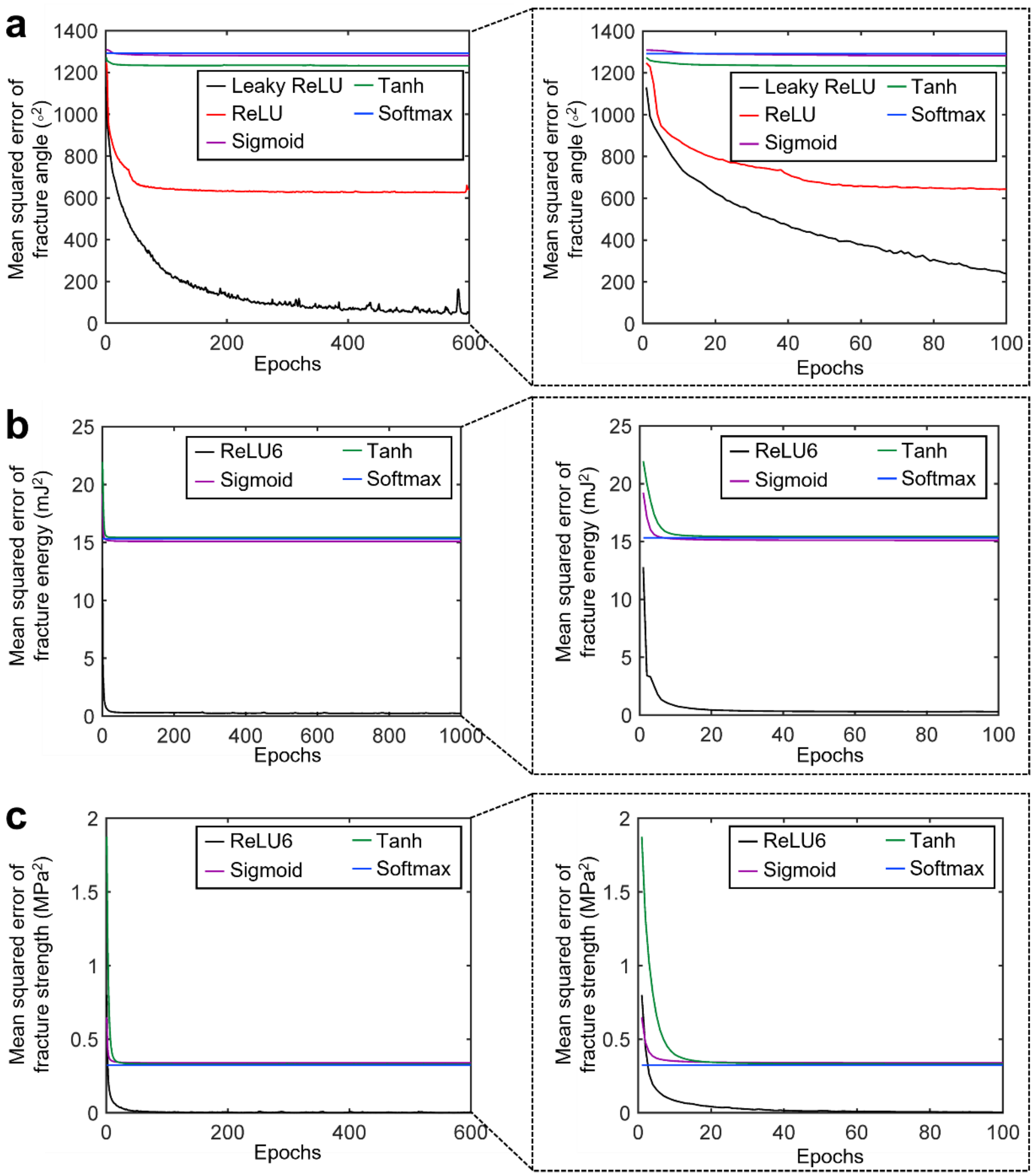

Supplementary Fig. 27. Loss functions using different activation functions. (a) Losses for fracture angle predictions. (b) Losses for fracture energy predictions. (c) Losses for fracture strength predictions. Note that ReLU represents rectified linear unit. Source data are provided as a Source Data file.

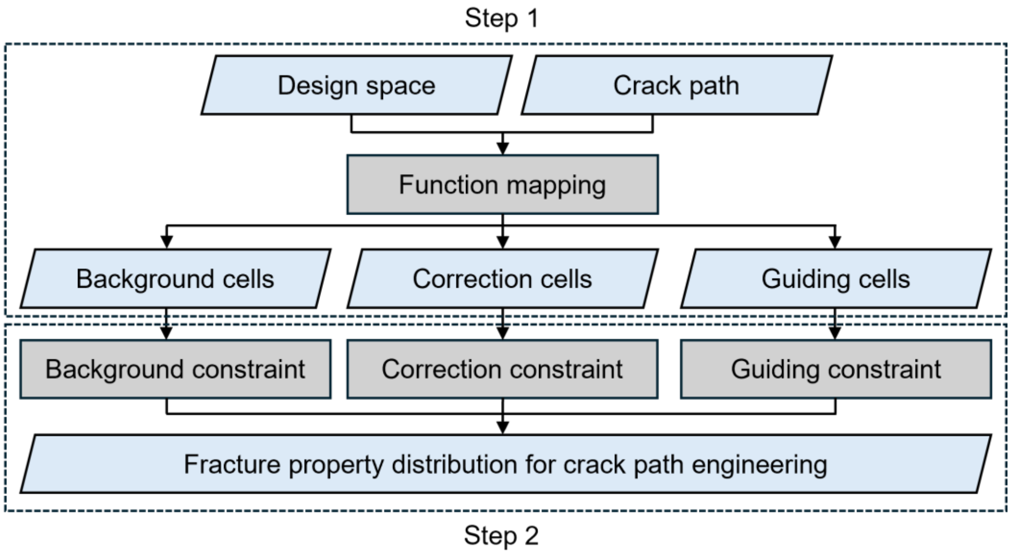

Supplementary Fig. 28. Flow chart of the crack path engineering algorithms.

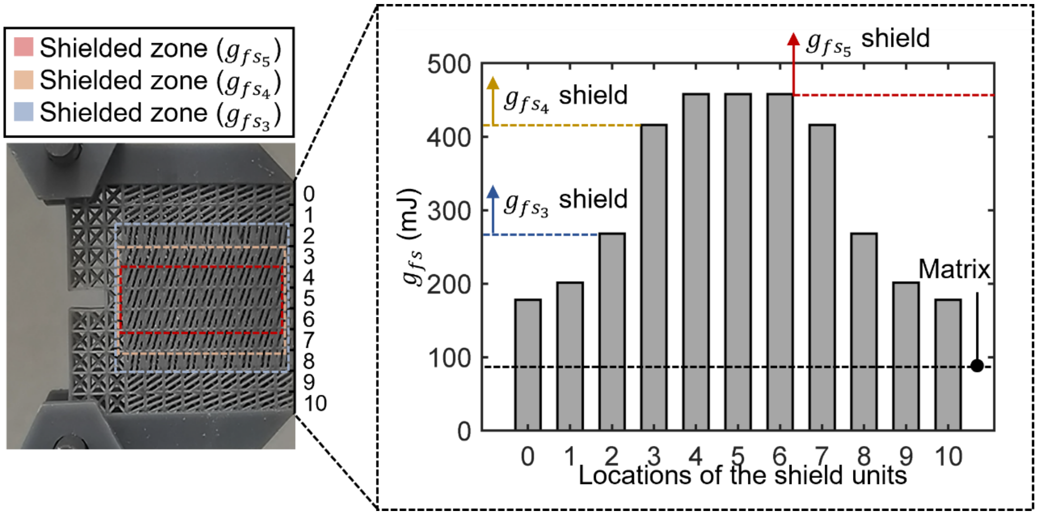

Supplementary Fig. 29. The shielding regions of the crack shielding specimen. g_{f_1}- g_{f_5} are the fracture energies of different shield units. Source data are provided as a Source Data file.

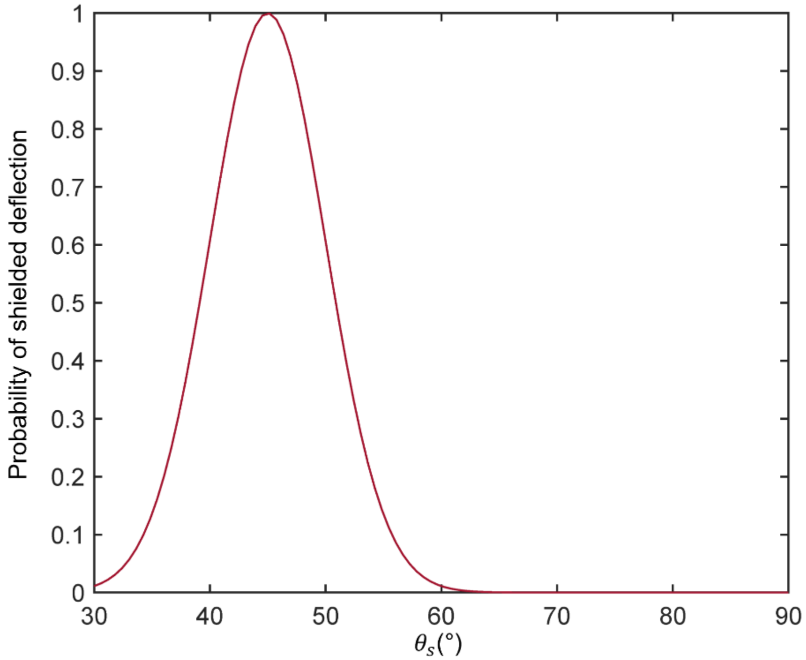

Supplementary Fig. 30. The possibility function of shielded deflection. θ_S is the shield angle. Source data are provided as a Source Data file.

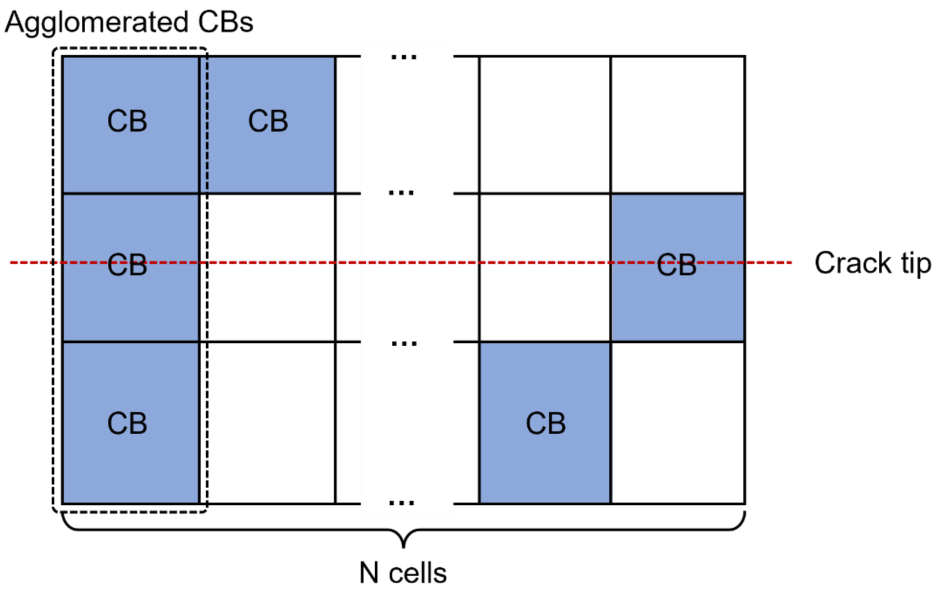

Supplementary Fig. 31. The schematics of the agglomerated crack bowing (CB) phases.

Supplementary Table 1. Summary of mechanical properties of parent material.

Specimen number	Modulus (GPa)	Ultimate tensile stress (MPa)	Ultimate tensile strain (%)	Test area (mm^2)
1	1.3	37.2	36.0	22.0
2	1.3	36.4	33.2	22.1
3	1.3	35.3	37.0	22.3
Average	1.3	36.3	35.4	22.1

Supplementary Table 2. Design data of damage-programmable (DP) cells, where PGS and MGS represents the primary guiding surface and minor guiding surface, respectively, T0 to T3 represents type-0 to type-3 DP cells.

Functional category	Type of DP cells	PGS angle 1	PGS angle 2	MGS angle 1	MGS angle 2	MGS angle 3	MGS angle 4
Spatially program the crack path	Guiding	Constrained by equation (S7-S13)					
	Correction						
	Background						
Construct toughening units	T0	Base cells without microfibers for comparison purposes					
	T1	90	90	0	0	90	90
	T2	80	80	0	0	90	90
	T3	Varies according to the pre-designed fracture angles					

Supplementary Table 3. Summary of experimentally observed bridging events, where S_B is the size of the bridging fibers, d_B is the bridging distances, A_B represent the statistical results of the bridging area per length of the bridging fibers.

S_B (Number of cells)	d_B (Number of cells)	A_B (Number of cell surfaces)	Bridged deflection (Yes/No)	Bridged cut-through (Yes/No)
3	1	0.67	Yes	No
	3	0.67	Yes	Yes
	5	1.00	Yes	No
	7	2.00	No	Yes
5	1	0.20	Yes	No
	3	0.30	Yes	Yes
	5	0.30	Yes	No
	7	0.30	No	Yes
7	1	0.21	Yes	Yes
	3	0.26	Yes	Yes
	5	0.17	Yes	No
	7	0.13	No	Yes

Supplementary Table 4. Summary of different specimens to evaluate the fracture of metamaterials.

Specimen type	2D planar fracture	3D fracture	2D compact tension
Number of cells	9×9×1	10×10×10	11×10×3
Functionality	Reveal the propagation of the crack on a 2D plane	Evaluates the fracture surface on a 3D design space	Obtain the fracture load-displacement curves and the resulted fracture energies

Supplementary Table 5. Optimized hyperparameters for the fully connected neural network designs of different training purposes, where ReLU represents rectified linear unit.

Training purposes	Number of hidden layers	Number of neurons in each hidden layer	Activation function	Learning rate	Number of training cycles
θ_f prediction	3	30	Leaky ReLU	0.01	600
σ_f prediction	3	40	ReLU6	0.1	600
G_f prediction	3	50	ReLU6	0.01	1000

References

1. Murphy KP. *Machine learning: a probabilistic perspective*. MIT press (2012).

2. Gao Z, Dong G, Tang Y, Zhao YF. Machine learning aided design of conformal cooling channels for injection molding. *Journal of Intelligent Manufacturing*, 1-19 (2021).

3. Qiao L, Liu Y, Zhu J. A focused review on machine learning aided high-throughput methods in high entropy alloy. *Journal of Alloys and Compounds* **877**, 160295 (2021).

4. Usuga Cadavid JP, Lamouri S, Grabot B, Pellerin R, Fortin A. Machine learning applied in production planning and control: a state-of-the-art in the era of industry 4.0. *Journal of Intelligent Manufacturing* **31**, 1531-1558 (2020).

5. Chaudry UM, Hamad K, Abuhmed T. Machine learning-aided design of aluminum alloys with high performance. *Materials Today Communications* **26**, 101897 (2021).

6. Chen G*, et al.* Machine-learning-assisted de novo design of organic molecules and polymers: opportunities and challenges. *Polymers* **12**, 163 (2020).

7. Abadi M*, et al.* Tensorflow: Large-Scale Machine Learning on Heterogeneous Systems, Software. *available from tensorflow org*, (2015).

8. Hochreiter S. The vanishing gradient problem during learning recurrent neural nets and problem solutions. *International Journal of Uncertainty, Fuzziness and Knowledge-Based Systems* **6**, 107-116 (1998).

9. Gao Z*, et al.* Data-driven design of biometric composite metamaterials with extremely recoverable and ultrahigh specific energy absorption. *Composites Part B: Engineering* **251**, 110468 (2023).

10. Huang W*, et al.* Multiscale toughening mechanisms in biological materials and bioinspired designs. *Advanced Materials* **31**, 1901561 (2019).

11. Gao Z, Li D, Dong G, Zhao YF. Crack path-engineered 2D octet-truss lattice with bio-inspired crack deflection. *Additive Manufacturing* **36**, 101539 (2020).

12. Srivastava A, Osovski S, Needleman A. Engineering the crack path by controlling the microstructure. *Journal of the Mechanics and Physics of Solids* **100**, 1-20 (2017).
