# Supplementary material for: Damage-programmable design of metamaterials achieving crack-resisting mechanisms seen in nature
Source: Nat Commun. 2024 Aug 27;15:7373. doi: 10.1038/s41467-024-51757-0 (PMC11349770; doi:10.1038/s41467-024-51757-0)
Supplement: Supplementary file 3 — Description of Additional Supplementary Files [file 41467_2024_51757_MOESM3_ESM.pdf]

Description of Additional Supplementary Files

File Name: Supplementary Movie 1

Description: The recording of the fracture processes and digital image correlation (DIC) analyses of the 3D and 2D damage-programmable (DP) specimens.

File Name: Supplementary Movie 2

Description: The recording of the fracture processes, normalized fracture load-displacement curves, and digital image correlation (DIC) analyses of the conventional comparison specimen and specimen with damage-programmable (DP) metamaterials with combined crack resistances.
